# Development of a tool for assessing awareness of consequences of suicide

**DOI:** 10.3389/fpsyg.2026.1736232

**Published:** 2026-02-16

**Authors:** Vanessa G. Macintyre, Daniel Pratt, Warren Mansell, Sara J. Tai

**Affiliations:** 1 Division of Psychology and Mental Health, School of Health Sciences, University of Manchester, Manchester, United Kingdom; 2 Manchester Academic Health Science Centre (MAHSC), Manchester, United Kingdom; 3 Greater Manchester Mental Health NHS Foundation Trust, Manchester, United Kingdom; 4 Curtin School of Population Health, Curtin enAble Institute, Curtin University, Perth, WA, Australia

**Keywords:** assessment tool, cognitive interviews, personal goals, suicidal behavior (SB), suicidal ideation (SI), suicide attempts (SA)

## Abstract

**Objectives:**

The ways in which individuals consider the potential impact of suicide on their life goals (i.e., consequences of suicide) may influence their suicidal ideation and/or behavior, but no measures exist for assessing awareness of consequences of suicide. A new measure is needed to ensure reliable and valid measurement which then enables accurate testing of hypotheses regarding consequences of suicide. The current study aimed to develop and refine a new tool for assessing awareness of potential consequences of suicide (Awareness Assessment Tool; AAT).

**Design:**

Multiple stages of AAT development involved analysis of qualitative data from two sets of interviews.

**Methods:**

Interviews with 12 participants who had contemplated or attempted suicide were analyzed using Reflexive Thematic Analysis (TA) to evaluate potential AAT items (Stage 1), followed by initial item development (Stage 2). Cognitive interviews to evaluate the AAT were then conducted with nine individuals with experience of suicidal ideation and/or behavior and analyzed using TA (Stage 3), followed by further AAT refinement (Stage 4).

**Results:**

The following themes were identified at Stage 1: “The interview as an acceptable and helpful experience” and “The relationship between one’s mindset and ability to remember experiences of suicide.” Stage 3 analysis generated the following themes: “Limitations of the AAT design”; “The AAT as an acceptable measure of a potentially helpful idea”; “The importance of accounting for failure to reach goals”; “Psychological processes regarding the potential impact of suicide captured by the AAT”; “The importance of assessing short-term goals.” In Stage 4, the phrasing and length of AAT items were refined, some items were added, and improvements were made to the interviewer instructions and prompts in the AAT.

**Conclusion:**

Patterns of responding to AAT items were highlighted and refinements were made to improve the AAT. Overall, the AAT was experienced as acceptable and sometimes helpful, with demonstrable potential for use in future research.

## 1 Introduction

Suicide is a public health priority, causing over 700,000 deaths per year (World Health Organization, 2023). The psychological processes underlying the progression from contemplating to attempting suicide are still not well-understood (Klonsky and May, 2014; Macintyre et al., 2021; May and Klonsky, 2016). Known psychological risk factors, such as hopelessness, perceived burdensomeness, and impulsivity, do not reliably predict suicide attempts (Franklin et al., 2017). This lack of explanatory power may be because each of these risk factors is only part of a much larger biopsychosocial interaction which is too complex to comprehend (Franklin, 2019). Furthermore, terms such as “thwarted belongingness,” which refer to key factors in prominent theories of suicide (e.g., O’Connor and Kirtley, 2018; Van Orden et al., 2010), aim to describe very precise emotional states but are inevitably imprecise due to the vagueness of natural language (Millner et al., 2020). As a result, there is ambiguity in these models of suicide in terms of the interactions specified between these psychological risk factors, and in the specified relationship between these factors and suicidal thoughts and behavior (Millner et al., 2020). Therefore, rather than investigating relationships between specific risk factors, it may be more beneficial to focus on core psychological processes which integrate risk factors such as hopelessness and thwarted belongingness (Macintyre et al., 2021). Insights into the role of these core psychological processes in suicide could advance the current knowledge on suicide and inform future interventions for the prevention of suicide (Macintyre et al., 2021).

A new theoretical framework for understanding suicide has recently been developed, which focuses on core processes hypothesized to underlie suicidal thoughts and behavior (Macintyre et al., 2021). Its development has been guided by Perceptual Control Theory (PCT; Powers, 1973), a transdiagnostic theory, which has proven valuable when applied to understanding and informing interventions for a variety of mental health issues, including psychosis and bipolar (Carey, 2008; Griffiths et al., 2019a,b; Healey et al., 2017; Mansell et al., 2014; Morris et al., 2016, 2018; Tai, 2009; Varese et al., 2017). PCT posits that individuals’ actions are attempts to control their perceptions, so that their experiences match a set of reference values, i.e., “just right” states, which are also referred to as the individuals’ goals (Carey, 2008; Powers, 1973). In PCT, the term goals encompasses everything from their most abstract long-term values (referred to as higher-level goals), such as their wish to be a kind person, to their most concrete short-term daily goals (referred to as lower-level goals), such as a goal to brush their teeth (Powers, 1973). These goals are structured in a hierarchy from their higher-level goals to their lower-level goals, and include unconscious physiological processes which are intrinsic for survival, such as maintaining a steady heart rate (Mansell, 2022; Powers, 1973).

When applied to understanding suicide, PCT posits that suicidal thoughts and behavior are a means of controlling one’s experiences (Macintyre et al., 2021). For example, someone who wants to experience relief from their distress might imagine that if they die by suicide, they will experience a sense of peace. However, the core psychological process hypothesized to underlie the progression from contemplating to attempting suicide is *limited awareness* of one’s goals (Macintyre et al., 2021). Macintyre et al. (2021) hypothesized that, regardless of which psychological risk factors are involved, individuals progress from contemplating suicide to attempting suicide when they have limited awareness of how their goals could be negatively impacted by suicide (i.e., *limited awareness of consequences of suicide*). Preliminary support for this hypothesis has been provided in two recent qualitative studies, in which the experiences of individuals who had contemplated or attempted suicide were explored (Macintyre et al., 2026a^1^; Wynford-Thomas et al., 2026^2^). In both studies, participants who had attempted suicide described experiences of having not fully considered potential consequences at the time of a suicide attempt. For example, some individuals who described their suicide attempt as very impulsive, only considered potential consequences after the attempt (Macintyre et al., 2026a; Wynford-Thomas et al., 2026). Others “minimized” consequences in their minds (e.g., “they’ll get over it and realize that they’re better off without me”) rather than more fully considering how suicide could impact on their goals, such as a goal to be there for their family (Macintyre et al., 2026a). In contrast, in both studies, participants who had contemplated suicide, but had not made a suicide attempt, described an acute awareness of reasons to avoid attempting suicide which were related to their goals (Macintyre et al., 2026a; Wynford-Thomas et al., 2026). These included fears of upsetting their family or going against their religion which viewed suicide as wrong (Macintyre et al., 2026a). However, this recent hypothesis on the role of limited awareness of consequences of suicide in the progression from contemplating to attempting suicide has not yet been tested quantitatively. Furthermore, no measures currently exist for assessing the theoretical construct of limited awareness which could be used to test the hypothesis. Therefore, in order to test the hypothesis posited by Macintyre et al. (2021) on the role of limited awareness in suicide, it is necessary to develop a reliable and valid tool for assessing limited awareness of one’s goals.

The new tool for assessing awareness of goals needs to assess multiple aspects of this awareness due to the assumptions of relevant theoretical literature. Firstly, PCT posits that individuals can have limited awareness of their higher-level goals which are motives for achieving certain goals (e.g., a goal to have a successful career as the motivation to graduate from university), or limited awareness of lower-level goals which are steps toward reaching these goals (e.g., a goal to complete university assignments) (Carey, 2006; Mansell, 2005; Powers, 1973). Therefore, the new assessment tool needs to assess respondents’ awareness of their motives for goals and awareness of the steps toward reaching their goals. Secondly, since having greater awareness of a goal is posited to involve a greater focus of attention on that goal (Carey, 2006, 2008; Mansell, 2005), this greater awareness would be likely to involve the following: the ability to access a goal (i.e., hold the goal in one’s mind’s eye); thinking of the goal frequently; an influence of the goal on one’s decisions. Therefore, it is essential that a new assessment tool assesses respondents’ ability to access goals, frequency of thoughts about their goals, and the influence of these goals on respondents’ decisions. In addition, individuals who have not fully considered the potential impact of suicide on their goals may be less likely to report that suicide would interfere with their goals. Conversely, they may be more likely to report that suicide would help with reaching their goals. Therefore, these potential aspects of awareness of goals and consequences of suicide also need to be included in the new assessment tool.

Since mental imagery is often powerfully connected to verbal processing, people often experience imagery when focusing their attention on one of their goals (Hackmann and Holmes, 2004). Furthermore, mental imagery plays a role in suicide since individuals can experience vivid images related to their suicidal ideation which can be distressing or comforting and may either deter or encourage suicidal behavior (Crane et al., 2012; Hales et al., 2011). Therefore, various characteristics of imagery (e.g., vividness, visual perspective) may be involved in awareness of goals and consequences of suicide and need to be included in the assessment tool. Lastly, limited awareness is posited to sometimes involve experiences of goal-related imagery as involuntary and may include a desire to suppress these images (Mansell, 2005; Mansell and Hodson, 2009), so these aspects of imagery also need to be assessed.

Cognitive interviews are a valuable tool in the development of new measures, such as questionnaires and surveys (Balza et al., 2022; Willis, 2011). During a cognitive interview, the interviewer uses various techniques which highlight potentially problematic items in the new measure, such as items which lack clarity or produce unwanted or irrelevant responses from individuals who are completing it (Balza et al., 2022; Willis, 2011). Given that the development of a novel assessment tool for assessing limited awareness is likely to pose challenges, the use of cognitive interviews is particularly essential. Since the new assessment tool is intended for use with a suicidal population in future studies, it is essential that the tool is easy to understand and complete, but that its items are considered safe and acceptable to potential respondents. The current study aimed to develop and refine the new assessment tool (referred to as the Awareness Assessment Tool; AAT) and to answer the following research questions: (1). How well does the AAT assess awareness of consequences of suicide?; (2). How acceptable is the completion of the AAT to participants? The study consisted of four stages (analysis, development, feedback and refinement), involving an iterative process of seeking feedback from participants on initial and later drafts of the AAT. This multi-stage process enabled feedback from participants to influence the AAT design, structure, and items throughout its development and refinement, ensuring that the AAT was acceptable and relevant to individuals with experience of suicidal thoughts and behavior.

## 2 Materials and methods

### 2.1 Design

The study involved analysis of qualitative interview data from a previous study (Macintyre et al., 2026a) (Stage 1) which informed the initial AAT development (Stage 2). Cognitive interviews were then conducted, with interview transcripts analyzed to identify patterns of responding in the AAT and to gain feedback on participants’ experiences of completing the tool (Stage 3). Lastly, refinements were made to the AAT based on data from the cognitive interviews (Stage 4).

During all qualitative analyses, the epistemological approach taken was of critical realism, which assumes that there is a single reality which is experienced by participants, but that their descriptions of that reality may vary depending on their culture or use of language (Braun and Clarke, 2022). This approach was considered to be most appropriate because we were interested in understanding participants’ shared experiences of answering questions on suicide and their personal goals, and assumed that their accounts reflected these shared experiences but would also be influenced by their language and culture.

### 2.2 Stage 1: Analysis of qualitative interview data from a previous study

In a previous study (Macintyre et al., 2026a), qualitative interviews which aimed to understand individuals’ experiences of awareness of consequences of suicide from a time when they contemplated or attempted suicide were conducted. During these interviews, following the set of questions regarding experiences of suicidal thoughts/behavior, feedback was sought on participants’ experiences of answering these suicide-related questions. The aim of this feedback was to inform the AAT development, highlighting the kinds of questions which would be suitable for inclusion in the AAT, therefore this feedback was analyzed during the current study. The methods involved in collecting and analyzing this qualitative feedback data are described in the following paragraphs.

#### 2.2.1 Participants and recruitment

Members of the public (aged 18+) from England and Wales with experience of contemplating or attempting suicide within the past 5 years were recruited by Macintyre et al. (2026a). Full details of the sample inclusion criteria and recruitment process are provided elsewhere (Macintyre et al., 2026a).

#### 2.2.2 Feedback questions on participants’ experiences of answering interview questions on awareness of consequences of suicide

Once participants from Macintyre et al. (2026a) had answered questions regarding their experiences of suicidal ideation/behavior, during the same interviews, they were asked the following questions to gain feedback on their experiences of the suicide-related interview questions: “How did you find the interview questions?”; “How well did you remember your experiences?”; “Which questions felt more straight-forward or difficult to answer?”; “Was there anything about the interview that you found less acceptable or less appropriate?”; “Was there anything that made you feel less safe?” (see Supplementary Data Sheet 1 for a copy of the interview topic guide from that study).

#### 2.2.3 Analysis

The feedback on suicide-related interview questions from the qualitative interviews in Macintyre et al. (2026a) was transcribed verbatim and analyzed using Reflexive Thematic Analysis (TA; Braun and Clarke, 2006, 2022) in the current study. TA is a flexible trans-theoretical method which can be used to generate a rich and detailed analysis of complex data (Braun and Clarke, 2006). TA was considered most suitable for the analysis since our focus was on understanding patterns of meaning across participants in terms of their experiences of answering questions about their personal goals and suicidal thoughts or behavior. Analysis included the following steps: familiarization with the data; preliminary coding; development of themes; refinement and naming of themes.

In the initial familiarization stage, the researcher re-read each of the transcripts multiple times. Preliminary coding then began, which involved highlighting and writing notes on potential codes which were relevant to the research questions. A combination of semantic and latent coding was used. Semantic coding involved generating codes based on what was directly expressed by participants, and latent coding involved generating codes based on implicit meaning in participants’ accounts (Braun and Clarke, 2022). Both types of coding were necessary since we assumed that the participants would be able to explicitly communicate their realities while giving the majority of their feedback, but that some of their statements could contain implicit meaning. A primarily inductive approach was taken, involving focusing on participants’ perspectives when generating codes (Braun and Clarke, 2022), since we primarily aimed to understand experiences of answering questions about personal goals and suicidal thoughts/behavior from the participants’ perspectives. After preliminary coding was completed, initial themes were developed using post-it notes. These themes were then refined and named following discussions with the research team.

The research team held expertise in suicide research and the application of Perceptual Control Theory (PCT) to mental health, which informed the coding. However, we aimed to be primarily data-driven in our approach to gain insight into participants’ experiences of answering questions on personal goals and suicidal thoughts/behavior from their perspectives.

### 2.3 Stage 1: results

#### 2.3.1 Participant demographics and relevant clinical history

Data from the previous qualitative study (Macintyre et al., 2026a), which informed the initial development of the AAT, was collected from a sample of 12 participants who provided feedback on their experiences of answering questions about suicide and their personal goals. The sample consisted of two separate groups: six individuals who had attempted suicide (attempters) within the past 5 years, and six individuals who had contemplated suicide only (contemplators) within the past 5 years. The participants had an age range of 19–48 years (*M* = 29.42) and included four males (33%) and eight females (66%). Two participants self-reported a diagnosis of bipolar disorder whereas other participants preferred not to state their diagnoses. Further demographic and relevant clinical history information about the sample recruited by Macintyre et al. (2026a) is shown in Table 1.

**TABLE 1:** Demographic and relevant clinical history information at the time of recruitment for participants recruited by Macintyre et al. (2026a).

Gender	Ethnicity	Diagnosis of mental health problem	Family members, relatives, or close friends died by suicide	Lifetime history of suicide attempt	Number of suicide attempts	Recency of last suicide attempt prior to recruitment for the study
Female	White British	Yes	Yes	Yes	12	3 years ago
Female	White British	Yes	Yes	Yes	2	1 year ago
Female	White Welsh	Yes	Yes	Yes	2	5 years ago
Male	White British	Yes	No	Yes	2	5 years ago
Female	White	Yes	Yes	Yes	1	1 year ago
Female	White	Yes	Yes	Yes	2	5 years ago
Male	White African	Yes	No	No	None	N/A
Female	Pakistani	Yes	No	No	None	N/A
Female	Mixed	Yes	Yes	No	None	N/A
Female	White British	No	No	No	None	N/A
Male	White British	Yes	No	No	None	N/A
Male	White	Yes	No	No	None	N/A

Adapted from Macintyre et al. (2026a).

#### 2.3.2 Themes identified from the Stage 1 qualitative data

When analyzing the qualitative feedback data on experiences of answering questions about suicide and personal goals from interviews by Macintyre et al. (2026a), the following themes were identified: “The interview as an acceptable and helpful experience” and “The relationship between one’s mindset and ability to remember experiences of suicide.” The first theme illustrates how participants described the interview on suicidal thoughts and/or behavior and their personal goals as an acceptable and sometimes helpful way of discussing their experiences. The second theme describes how participants’ ability to remember their experiences (when answering questions about experiences of suicide) depended on how similar their mindset at the time of the interview was to their mindset during their previous experiences of suicidal thoughts/behavior.

##### 2.3.2.1 The interview as an acceptable and helpful experience

In the first theme, participants expressed that despite suicide being a “touchy” and potentially difficult subject, they found the interview questions on their experiences of suicidal thoughts/behavior and their personal goals acceptable:

*“It’s a touchy subject as it is so yeah but it’s – I mean personally, I found the way the questions were asked to be very acceptable*… *Some people might take it differently, it’s very difficult to gauge everybody, and what different audiences take things differently so I wouldn’t be able to comment on other people but yeah. I found it acceptable for me.” (Participant 10)*

*“I thought it would be harder and it’s not.” (Participant 1)*

Furthermore, some participants experienced the interview questions as positive and helpful, since the interview provided them with an opportunity to talk about their experiences:

*“I think it was really helpful to talk about it.” (Participant 11)*

*“To be honest it was quite nice to talk about it in a way, cause I never do. It was quite, I don’t know, feels healthy.” (Participant 12)*

For some participants, this enabled them to draw connections to make further sense of their own experiences:

*“I feel like it made me like aware of things that I hadn’t really thought about before, which was like helpful actually I think, like you know it made me draw connections between things that I hadn’t really thought about before… It really brought out to me how much more I value my life now… I thought they were really good questions.” (Participant 2)*

Moreover, one participant particularly appreciated the opportunity to discuss how their mental imagery affected their experiences of contemplating suicide, and highlighted the importance of being asked about mental imagery:

*“I found it interesting that you asked about the visual because, in the images, because no one’s ever asked me that before. Ever… But some of those images have really affected how I am*,

*you know, my mental state that day… When it happens the rest of the day is ruined because that’s all I’ve got stuck up there… It is important what you visualize actually… No one really asks you what you visualize.” (Participant 1)*

##### 2.3.2.2 The relationship between one’s mindset and ability to remember experiences of suicide

When the participants were asked how well they remembered their experiences of contemplating/attempting suicide, they described the need to put themselves back into the mindset which they had experienced while suicidal, in order to remember:

*“I think it took a few minutes of initially going back to that period to actually really analyze what it was like and what feelings I was experiencing – so actually going back and going back into that frame of mind… It’s like sort of transporting myself back to that period… and then remembering what I was thinking and feeling.” (Participant 7)*

For some participants, this was difficult due to the kinds of thoughts they experienced while suicidal, and because they were no longer in the same mindset at the time of the interview:

*“Very very difficult*… *It’s going to be difficult to remember, especially when your thoughts are so clouded at those moments in time.” (Participant 10)*

*“These spur of the moment sort of just impulsive thoughts – I don’t have them any more so I can’t really connect to them.” (Participant 4)*

*“It’s weird because it’s just such a different emotional state that it kind of feels like it isn’t me that I’m talking about. It kind of feels like I’m telling you about something I’ve read in a book about someone else, because I can’t really relate to the state of mind that I was in at that point, when I’m not in it… I really see myself then as a different person to me now, so it really felt like I’m talking about a different person.” (Participant 2)*

However, for participants who still experienced passive suicidal thoughts at the time of the interview (despite not being actively suicidal), their experiences of contemplating/attempting suicide were easier to remember as they could relate more easily to that mindset:

*“I would say I do still remember it and possibly even cause like I mention now in terms of, like those thoughts aren’t completely gone so they’ll still likely be the same, but it’s just in the past it’d obviously just be a lot more intense in times when I’ve been a lot more distressed.” (Participant 8)*

*“[How easy was it to remember like these experiences?] It’s quite easy”. (Participant 9)*

### 2.4 Stage 2: initial development of the Awareness Assessment Tool (AAT)

Following analysis of the feedback data from Macintyre et al. (2026a), the initial development of the AAT was informed by this feedback alongside relevant literature on suicide, mental imagery, and applications of PCT to understanding mental health (relevant theoretical assumptions are displayed in Table 2). The process of developing AAT items involved the following steps: (i) generation of theoretical assumptions about the experience of awareness of consequences of suicide based on relevant literature (see Table 2); (ii) initial decisions regarding how best to assess aspects of the experience of awareness of consequences of suicide (e.g., structure and format of the AAT); (iii) generation of individual AAT items, involving rephrasing items from the interview topic guide used by Macintyre et al. (2026a) and creation of some new items; (iv) discussions within the research team about the phrasing and scoring of these items and subsequent refinements; (v) examination of feedback data from Macintyre et al. (2026a) for feedback on suicide-related questions which should be added to, removed from, or retained within the AAT; (vi) discussions by the research team about potential updates to the AAT based on this feedback, and subsequent changes if applicable. Several iterations of the AAT were designed, discussed, and refined by the research team before a version suitable for use in Stage 3 was finalized. Although these refinements were not based on fixed empirical criteria, each involved a review of the latest AAT version by the research team and amendments to ensure that: (i) the AAT assesses all theoretically relevant constructs (outlined in the introduction and Table 2); (ii) its format and structure allow ease of completion; (iii) interviewer instructions are sufficient; (iv) items avoid leading questions; (v) items are clearly phrased in lay terms; and (vi) items include appropriate Likert-type scales where applicable.

**TABLE 2:** Indices of awareness and the theoretical assumptions or findings which the indices of awareness are based on.

Index of awareness	Relevant assumptions or findings from literature
Respondents’ ability to describe motives for their goals	PCT posits that individuals can have limited awareness of higher-level goals (i.e., their underlying motives for reaching certain goals), which can be distressing (Carey, 2006; Mansell, 2005; Powers, 1973) and may influence suicidal behavior (Macintyre et al., 2021). In a study investigating depression, symptoms were highest in individuals with high levels of ambivalence (explained as conflict between higher-level motivational goals), and distress is posited to persist when conflicting motivational goals are outside of conscious awareness (Alsawy et al., 2014; Kelly et al., 2011). An example was provided by authors of the same study of a woman experiencing ambivalence over a goal to try for a baby, who “may feel this way because her goals to “please my parents” and “be independent” drive her in opposing directions when it comes to deciding whether to pursue the goal” (Kelly et al., 2011, p533).
Respondents’ ability to access goals in their minds	Greater awareness of a goal would involve the ability to access the goal (i.e., hold the goal in one’s mind’s eye), frequent thoughts about the goal, and an influence of the goal on decisions (Carey, 2006, 2008; Mansell, 2005). These assumptions are based on recommendations for PCT-informed clinical work, which aims to increase clients’ awareness of their goals to reduce goal conflict (Alsawy et al., 2014). Clinicians are advised to explore and increase clients’ awareness of their goals by asking questions which facilitate a focus on their goals in the present moment, such as questions about clients’ ability to focus on and visualize goals (Alsawy et al., 2014). Therefore, similar questions were included within this index of awareness.
Respondents’ ability to describe steps to reach goals	PCT posits that individuals can have limited awareness of lower-level goals (i.e., the steps toward reaching certain goals), which can cause distress (Carey, 2006; Mansell, 2005; Powers, 1973). For example, individuals with an exclusive focus on higher-level goals may perceive their goals as being achieved less frequently due to a lack of attention toward short-term goals (Mansell, 2005). It is posited that difficulties in generating specific concrete details in imagined events are due to limited awareness of short-term goals, and this limited awareness may explain findings that suicidal individuals experience problem-solving difficulties (Macintyre et al., 2021; Schotte and Clum, 1987).
Strength of respondents’ beliefs that goals could be reached if they died by suicide	Assumptions were made by authors of the current study, based on a PCT-informed framework for understanding suicide (Macintyre et al., 2021), that individuals who have not fully considered the potential impact of suicide on their goals may be less likely to report that suicide would interfere with their goals.
Strength of respondents’ beliefs that suicide would help with reaching goals	Assumptions were made by authors of the current study, based on a PCT-informed framework for understanding suicide (Macintyre et al., 2021), that individuals who have not fully considered the potential impact of suicide on their goals may be more likely to report that suicide would help with reaching their goals.
Vividness of goal-related mental imagery	Studies have shown that experiences of imagery are frequently linked to the self and specific goals (Mansell and Hodson, 2009; Reid, 2009). For example, in a study examining imagery in clinical and non-clinical participants, almost 90% of imagery reported by participants was associated with at least one goal (Reid, 2009). PCT posits that imagery occurs as a means of regaining control of one’s experiences by mentally simulating goal achievement (Huddy and Mansell, 2023), and this is consistent with previous literature on mental simulation (D’Argembeau and Mathy, 2011). Furthermore, the vividness of goal-related imagery may reflect awareness of goals. Thought/imagery suppression is one way in which limited awareness may occur (Macintyre et al., 2021), and imagery has been found to be more vivid for individuals who do not suppress the imagery (D’Argembeau and Van der Linden, 2006). In addition, suicidal individuals report vivid suicide-related images which can influence their mood and their suicidal thoughts/behavior, and which are related to important aspects of their lives (i.e., their goals) such as their relationships with others (Crane et al., 2012; Hales et al., 2011; Lawrence et al., 2023). For example, one participant imagined their family finding their body and was subsequently deterred from ending their life (Hales et al., 2011). Therefore, the inclusion of an index of awareness for assessing vividness of goal-related imagery was considered important.
Broadness of visual perspective of goal-related mental imagery	This index was included for the same reasons as 6) Vividness of any goal-related mental imagery experienced by respondents. In addition, in imagery literature it has been hypothesized that the visual perspective of an imagined event is related to perceptions of that imagined event (Libby and Eibach, 2011; Niese et al., 2024). Viewing events from the observer (third-person) perspective (as opposed to first-person perspective) is hypothesized to involve viewing the imagined event in the context of other aspects of one’s life as opposed to merely focusing on momentary sensory experiences (Libby and Eibach, 2011; Niese et al., 2024). Therefore, a broader visual perspective of imagery may indicate greater awareness of a goal.
Voluntariness/involuntariness of goal-related mental imagery	This index was included for the same reasons as 6) Vividness of any goal-related mental imagery experienced by respondents. In addition, studies have shown that imagery related to one’s goals is experienced as intrusive and involuntary if the imagery is negative (Hackmann and Holmes, 2004; Mansell and Hodson, 2009; Reid, 2009). For example, a qualitative analysis of experiences of individuals with bipolar disorder indicated that intrusive images were consistently related to participants’ negative self-concepts (e.g., feelings of worthlessness and hopelessness) (Mansell and Hodson, 2009). Another study examining imagery and goals revealed that the majority of intrusive images were associated with a goal to avoid a particular situation (i.e., avoidance goals) (Reid, 2009). Involuntary imagery has been explained as occurring due to experiences of conflict between one’s goals (Huddy and Mansell, 2023; Mansell, 2005). For example, “A person who holds the goal of achieving safety would be more likely to perceive a mental image of a hostile face as involuntary because it conflicts with this goal” (Huddy and Mansell, 2023, p69).
Willingness to experience goal-related mental imagery	This index was included for all of the same reasons as 8) To what extent respondents perceive/perceived goal-related mental imagery as involuntary. In addition, if imagery is experienced as involuntary, individuals may be motivated to suppress the imagery to avoid experiencing it, which can lead to more limited awareness of the goals that are related to the imagery (Macintyre et al., 2021; Mansell and Hodson, 2009).

PCT, Perceptual Control Theory.

### 2.5 Stage 2: results

Nine different aspects of the experience of awareness of consequences of suicide were identified from relevant literature (referred to as *indices of awareness* when assessed using the AAT) which subsequently guided the development of AAT items in an initial draft. These indices of awareness and their supporting literature are presented in Table 2. Analysis of the feedback from Stage 1 indicated that the kinds of AAT questions used to assess these indices of awareness would be experienced as acceptable, since similar questions were considered acceptable during the interviews by Macintyre et al. (2026a). In addition, although not a theme, some of the feedback data indicated potential advantages of including questions about mental imagery. Since this feedback supported the inclusion of existing AAT items and did not identify any additional aspects of awareness of consequences of suicide not already included in the AAT, no further changes were made following this feedback.

### 2.6 Stage 3: cognitive interviews

Following initial development of the AAT (Stages 1 and 2), a new sample were recruited to participate in cognitive interviews, with the aim of gaining feedback on participants’ experiences of completing the AAT. The methods involved in recruitment and cognitive interviews with this new sample are described in the following paragraphs.

#### 2.6.1 Participants and recruitment

In Stage 3, members of the public from England and Wales were recruited from mental health and suicide charities. University of Manchester students were also recruited using the University of Manchester SONA system, an online platform which enables University of Manchester undergraduate psychology students to gain course credits as compensation for taking part in research studies. All participants had recent experiences of mental health issues and/or suicidal thoughts or behavior.

Participants were included in the study if they met the following criteria: aged 18 or over; had either contemplated or attempted suicide within the past 5 years; were registered with an NHS GP; intended to reside in the UK for 6 months after participating in the study (so that any suicide risk issues could be followed up with their GP and/or mental health team).

When recruiting from mental health charities, the researcher asked them to share study information with members, who then contacted the researcher to express interest. Participants recruited via the University of Manchester SONA system signed up for a study timeslot and were then contacted. In both cases, the researcher emailed further details and invited questions before screening for eligibility. Participants confirmed past suicidal thoughts or attempts within 5 years and met criteria including not actively planning suicide, being registered with an NHS GP, and residing in the UK for six months. Those with language, communication, or cognitive difficulties were excluded. After consent, participants provided GP or mental health team contact details, who were consulted for any concerns before interviews. Participants would have been excluded if their GP or mental health team had raised concerns that could not be resolved, although no such concerns were reported for any participant.

#### 2.6.2 Measures

##### 2.6.2.1 Awareness Assessment Tool (AAT)

The AAT is an assessment tool which is administered by an interviewer and aims to assess an individual’s awareness of how suicide could impact on their life goals or values (i.e., awareness of consequences of suicide). This is achieved by assessing various potential indicators of respondents’ awareness of their goals and how these goals could be impacted upon by suicide. These are referred to as indices of awareness and were devised based on theoretical assumptions about aspects of awareness of consequences of suicide that need to be assessed, which are described in Table 2. (See Supplementary Data Sheets 2, 3 for a copy of the initial AAT version used in the current study and its corresponding answer booklet).

The AAT consists of two halves, the first of which asks about respondents’ present experiences (i.e., the Present Time Experiences Section), and the second of which asks about their experiences at the time they most recently contemplated suicide (i.e., the Contemplating Suicide Section). These two sections allow comparison of respondents’ awareness of consequences of suicide between the time of AAT completion and when they most recently contemplated suicide in future studies using the AAT.

In each section, respondents are then asked to list three important goals which they focused on at that time (past and when contemplating suicide), and to rate the importance of these goals from 0 to 10, followed by a series of questions about each goal. Respondents are advised that they can list the same goals in both halves (the Present Time Experiences Section and Contemplating Suicide Section), or they can be different goals, depending on their experiences.

The series of questions in each half of the AAT aims to assess the following indices of awareness:

- Respondents’ ability to describe motives for their goals
- Respondents’ ability to access goals in their minds
- Respondents’ ability to describe steps to reach goals
- Strength of respondents’ beliefs that goals could be reached if they died by suicide
- Strength of respondents’ beliefs that suicide would help with reaching goals
- Vividness of goal-related mental imagery
- Broadness of visual perspective of goal-related mental imagery
- Voluntariness/involuntariness of goal-related mental imagery
- Willingness to experience goal-related mental imagery.

After completing the Present Time Experiences Section only, respondents are asked questions regarding the way in which they thought about the goals they listed in that section, when they last contemplated suicide. For example, for each goal listed for the present time, they are asked, “To what extent did it influence your decisions around the time that you most recently contemplated suicide?”

The AAT items follow a flowchart format (see Supplementary Data Sheets 2, 3 for a copy of the interviewer flowchart and instructions) so that if the respondent is unable to answer certain questions (e.g., “Thinking about your current situation right now, how would you reach this goal?”), they are asked other questions which they may be more able to answer (e.g., “What gets in the way of you reaching this goal?”). The AAT collects a mixture of quantitative data (most of which is rated on Likert-type scales, e.g., 0–5), and qualitative data (e.g., responses describing the steps required to reach a particular goal). However, the AAT items were not scored during the current study despite having been presented to participants, since the current study aimed to develop and refine the AAT rather than collecting quantitative data on awareness of consequences of suicide.

##### 2.6.2.2 Adapted positive and negative affect schedule within the AAT

So that participants’ moods during each time point (present and when previously contemplating suicide) can be assessed prior to completion of the goal-related questions in future studies, the beginning of each AAT section contains an adapted version of the Positive and Negative Affect Schedule (PANAS; Watson et al., 1988). Respondents’ mood may affect their awareness of consequences of suicide (Macintyre et al., 2021), so assessing mood in the present and when previously contemplating suicide could provide a more comprehensive understanding of respondents’ experiences in future research. The adapted PANAS contains 10 items assessing positive emotions and 10 items assessing negative emotions, and participants are asked to rate to what extent (“Strongly Disagree” to “Strongly Agree”) they are/were experiencing that emotion. In the current study, the adapted PANAS was presented to participants within the AAT to gain feedback on their overall experience of completing the AAT. However, quantitative data was not collected using the adapted PANAS at this early development phase of the AAT. This was because the purpose of the current study was to develop and refine the AAT, rather than collecting quantitative data on participants’ awareness of consequences of suicide or moods while contemplating suicide (which could be collected in future research).

##### 2.6.2.3 Cognitive interview topic guide

The topic guide for the cognitive interviews was informed by guidance from relevant literature on the use of cognitive interviews to refine newly developed questionnaires and measures (Willis, 2011), and aimed to evaluate the AAT items. It included two cognitive interviewing techniques, the “think-aloud” technique and “verbal probes” (Willis, 2011), to ensure that any potential problems with the AAT items were identified, such as the following: misunderstandings in how items should be answered; lack of clarity in the phrasing of items; difficulties in remembering the information required to respond to the items.

The “think-aloud” technique involves asking participants to describe everything that comes to mind as they are responding to each item, thereby revealing the thought processes involved in answering. This revealed any difficulties in responding and highlighted occasions where participants answered questions in ways which were unexpected by the researcher. The “verbal probes” allowed the researcher to evaluate the AAT items by gaining direct feedback from participants, and included questions such as the following: “Was that easy or hard to answer?”; “How did you arrive at that answer?”; “How well do you remember that?”; “Can you repeat the question I just asked in your own words?” In addition, in order to assess the acceptability and safety of the interview questions, when participants responded to some of the AAT items, particularly those related to suicide, they were asked questions such as “How appropriate did you feel that question was?” and “Was there anything about that question that made you feel less safe?”

#### 2.6.3 Data collection

Prior to the interviews, participants self-reported demographic and relevant clinical history data via email. The cognitive interviews lasted between 60 and 90 min and took place either in-person in a private room at the University of Manchester campus or remotely using Zoom software (Version 5.8.0, Zoom Video Communications, Inc, San Jose, California, United States). All interviews were audio-recorded using either Zoom or an encrypted audio-recording device.

When the researcher met with a participant, the participant was briefed with information about the study, including the kinds of questions they would be asked and the cognitive interview techniques which would be involved. During this process, they were given an opportunity to practice the “think-aloud” technique by answering a question which was unrelated to suicide. Next, participants were asked further clinical history questions (about the recency and number of times they had attempted or contemplated suicide) which were too sensitive to be asked via email. Participants who had attempted suicide were asked for information about their attempts, whereas participants who had contemplated suicide only were asked for information about their previous suicide contemplation. However, no participants were asked for details about experiences of both suicide attempts and contemplation. This was to ensure that sensitive questions about personal history were kept to a minimum, since feedback from a previous study (Macintyre et al., 2026a) indicated that some individuals may feel uncomfortable answering these kinds of questions.

The cognitive interview itself then began, during which the researcher administered the AAT. The researcher asked each AAT item and the participant responded verbally, and the researcher recorded the participant’s responses in an answer booklet. For each item, participants were asked to use the ‘think-aloud’ technique as they responded (i.e., to talk through their answers as they responded to the item rather than planning their answer and then stating it), which revealed their thought processes and patterns in responding to AAT items. If a participant appeared to struggle with answering an AAT item or if their thought process while responding indicated a lack of clarity in the way the item was phrased, the researcher used verbal probes to further evaluate the item. These revealed weaknesses of the AAT item and potential ways it could be improved. In addition, the researcher used verbal probes to evaluate items which were anticipated to be more difficult to answer, and asked participants questions on the acceptability and safety of items that were anticipated to be potentially distressing. Throughout the interview process, the researcher regularly “checked in” with participants to ensure that they were not experiencing any undue distress due to the interview.

Between completion of the Present Time Experiences Section and Contemplating Suicide Section of the AAT, participants were offered a short comfort break. The aim of this break was to provide a distraction to minimize the influence of participants’ responses from the Present Time Experiences Section on their responses in the Contemplating Suicide Section. Once both sections of the AAT had been completed, participants were asked for brief feedback about the tool’s overall acceptability. Participants were then asked to complete a short positive mood induction task consisting of two questions about their personal strengths, which aimed to counteract any potential distress experienced while answering suicide-related questions. Participants were then granted course credits (where applicable) or offered £5 (via a bank transfer arranged by the university finance department) as a token of appreciation for participating.

#### 2.6.4 Analysis

The feedback from the cognitive interviews was analyzed using Reflexive Thematic Analysis (TA; Braun and Clarke, 2006, 2022), following the same steps as the Stage 1 analysis of interviews from Macintyre et al. (2026a): familiarization with the data; preliminary coding; development of themes; refinement and naming of themes. TA was also chosen for this analysis due to our focus on understanding patterns of meaning across participants in terms of their experiences of completing the AAT.

This analysis involved the same process as the Stage 1 TA, i.e., applying a combination of semantic and latent coding with a primarily inductive approach (all of which are defined and explained in section 2.2.3 “Analysis”), since we had the same assumptions for this analysis. Both semantic and latent coding were used since our assumption was that participants would explicitly communicate their realities while providing feedback about the AAT, but that some descriptions of participants’ experiences could contain implicit meaning. The potential for participants’ statements to contain implicit meaning was particularly anticipated when participants completed the ‘think-aloud’ technique, since their explanation of their response to an AAT item could indicate a misunderstanding of that item. A primarily inductive and primarily data-driven approach was considered most suitable since our focus was on understanding experiences of completing the AAT from participants’ perspectives. However, similarly to Stage 1, it is possible that the research team’s expertise on suicide research and Perceptual Control Theory (PCT) may have influenced this analysis.

#### 2.6.5 Ethical approval

Ethical approval for the study was granted by the University of Manchester Full Research Ethics Committee (UREC reference: 2022-13005-25353). Data were anonymized at the point of transcription.

### 2.7 Stage 3: results

#### 2.7.1 Participant demographics and relevant clinical history

Nine participants aged 19–46 (*M* = 23.67) were recruited for the Stage 3 cognitive interviews, consisting of eight females and one male, all of whom had either contemplated or attempted suicide within the past 5 years. There was some overlap between this sample and the sample recruited by Macintyre et al. (2026a). Demographic and relevant clinical history data from the sample recruited for Stage 3 is displayed in Table 3.

**TABLE 3:** Demographic and relevant clinical history information at the time of recruitment for participants who were recruited for Stage 3.

Ethnicity	Diagnosis of mental health problem	Family members, relatives, or close friends died by suicide	Lifetime history of suicide attempt	Number of suicide attempts	Recency of last suicide attempt prior to recruitment for the study	Number of times they had contemplated suicide	Recency of last time they contemplated suicide prior to recruitment (if no suicide attempt within the past 5 years)
British White	Yes – preferred not to state diagnosis	No	Yes	2	12 months ago	Info not provided	Info not provided
White British	Yes – preferred not to state diagnosis	Yes	Yes	9	5 years ago	Info not provided	Info not provided
Mixed (White British and Asian)	Yes – preferred not to state diagnosis	Yes	No	N/A	N/A	3	8 months ago
German/Pakistani	Yes – preferred not to state diagnosis	No	No	N/A	N/A	1	4 months ago
White European	Yes – preferred not to state diagnosis	No	No	N/A	N/A	10	3 months ago
Chinese	Yes – preferred not to state diagnosis	No	No	N/A	N/A	Regularly 5 years ago then on 1–2 days per week more recently	2 weeks ago
Pakistani	No	No	No	N/A	N/A	Regularly	1 year ago
White British	Yes – preferred not to state diagnosis	No	No	N/A	N/A	Often, depending on the situation	1 week ago
White British	Yes – preferred not to state diagnosis	No	No	N/A	N/A	Multiple times	2 years ago

For reasons explained in section “2.6.3 Data collection,” information was not provided on suicide contemplation if it had already been provided for suicide attempts.

#### 2.7.2 Themes identified from analysis of the cognitive interviews in Stage 3

The following themes were identified from the transcripts of the cognitive interviews: “Limitations of the AAT design”; “The AAT as an acceptable measure of a potentially helpful idea”; “The importance of accounting for failure to reach goals”; “Psychological processes regarding the potential impact of suicide captured by the AAT”; “The importance of assessing short-term goals.”

##### 2.7.2.1 Limitations of the AAT design

While completing the AAT items, participants highlighted several design flaws which needed to be addressed, such as the phrasing and length of items, and the prompts provided by the interviewer. For most participants, this included a lack of clarity in the wording of certain items, such as an item which aimed to assess their ability to access a goal in their minds (“How able are you to bring this goal to mind?”). When responding to this item, participants expressed confusion at what was being asked of them or misunderstood the question:

*“I don’t – that question is a bit confusing to me, how able are you to bring to mind as in like – I don’t really know what that means. Like, how – what, just thinking of the goal or –” (Participant 3)*

*“How able am I to come up with the goal?” (Participant 5)*

Some limitations also involved items that did not provide enough potential response options in certain multiple-choice items. For example, when participants were asked whether they experienced goal-related mental imagery from the first-person or third-person perspective, some participants indicated that they experienced it as both:

*“Well, when it’s like – when it’s feeling it’s more through my eyes or like my senses, but then when it’s – when I’m like envisioning myself graduating, it’s third person, so it varies.” (Participant 7)*

In addition, feedback was given that the acceptability of the suicide-related questions could be improved by providing warning in advance that respondents would be asked about suicide:

*“I thought the ones with the S word, I thought, like, wow, that’s a bit – that just seems a bit extreme, mentioning it in that context*… *I think if you possibly to say to people, “I’m going to ask a few questions now, but I’ll mention that word*… *just like answer the questions as honestly as you can.” Maybe put that in before those S words.” (Participant 9)*

##### 2.7.2.2 The AAT as an acceptable measure of a potentially helpful idea

Overall, the participants experienced the AAT as an acceptable and safe measure and described how from their perspective, it was fine to complete and sometimes even a positive experience:

*“Yeah, it was good, I thought it was good ‘cos it rationalizes a lot of irrational feelings that you have at those periods of time, where you’re like contemplating suicide and stuff. I think actually putting it into words is pretty helpful.” (Participant 2)*

*“Yeah, it was fine really*… *I think there’s no shame in having those thoughts, and I think it’s important to think about it, and think back and compare to how you think now, you know*…. *I thought it was actually quite a nice experience, weirdly enough.” (Participant 4)*

*“It’s very, very good questions. I did enjoy reading them through, so.” (Participant 8)*

##### 2.7.2.3 The importance of accounting for failure to reach goals

The third theme describes the kinds of comments made by participants, while completing the AAT, indicating a potential relationship between perceived failure to reach goals and suicidal thoughts. This finding highlighted the necessity of adding an AAT item asking about failure to reach goals so that any potential impact of failure to reach a goal can be accounted for during assessments using the AAT. A number of participants, when responding to the item “If you died by suicide, how much would it help with achieving this goal?” described how it could either help with reaching the goal itself, or help them to stop worrying about failing to reach the goal:

*“In a way it would help [with a goal to have more control over their stress] in the sense that I wouldn’t have to stress about – there’s nothing that would really be stressing me out.” (Participant 4)*

*“People will just be like, “oh this person’s committed suicide,” nobody’s going to care what grades am I – was I getting [regarding a goal to achieve good grades]*…*So it’s like no one would be focusing on that*… *the goal is not important anymore.” (Participant 5)*

Others, when answering the item “If you died by suicide, how much would it interfere with the achievement of this goal?” described how not reaching the goal had led to their suicidal thoughts:

“…*the stress of, like, thinking about it [trying to achieve the goal] and thinking about it over and over again, it’s like, well, like it can lead me to feel the stress, which can then lead me to thinking more about suicide.” (Participant 3)*

*“I guess in those situations you’re so – for me at least, so sick of being sad all the time, I want to improve my wellbeing somehow, that’s like my goal, right, is to make myself happier, to do anything to do that, to get me out of my funk. So, you know, I guess suicide is one of those things, though it’s just not a very good thing.” (Participant 2)*

##### 2.7.2.4 Psychological processes regarding the potential impact of suicide captured by the AAT

Findings indicated various responses to the items “If you died by suicide, how much would it interfere with the achievement of this goal?” and “At the time that you most recently contemplated suicide, how much did you feel that it would interfere with the achievement of this goal if you died by suicide?”. This variety of responses may reflect various psychological processes captured by the AAT, which was considered a strength of the AAT since its purpose is to assess psychological processes which may underlie or affect awareness of consequences of suicide. Some participants described how much suicide would interfere with some of their goals being reached:

*“I think that it would interfere a lot… I know that it’d destroy them (family members), because like I’ve had this conversation before with them… I talked to my sister about this when I was really struggling, and she was like, “You can’t kill yourself.” Because I would kill her – she’d kill herself, because we’re dependent on each other… So like, it wouldn’t be the best if I did that.” (Participant 1)*

Other participants indicated that the goal could either potentially still be reached or would no longer be important if they died by suicide:

*“If I was going to kill myself, it would be a case of I can’t do it on the way down to the doctors because my booster is there… And that was my goal at the time, to have the booster. So, I’d have had the booster first and then, it could have gone either way with me. It would be a case of, I will have this booster and then I will kill myself, because I achieved my main goal. So, this is my reward, I can now kill myself.” (Participant 8)*

“…*They’d like have the like memory of me, you know. So like it’s still possible for us to be close [regarding a goal to maintain a relationship with family] even after I’ve died, so yes, I think*… *If anything, it might – like me dying might bring up – might make them love me more, I don’t know.” (Participant 7)*

*“So it wouldn’t interfere a lot because the goal wouldn’t exist anymore*… *Like after you die, there’s nothing you need to worry about, all of the goals and stuff.” (Participant 5)*

##### 2.7.2.5 The importance of assessing short-term goals

In the fifth theme, participants described how when they were feeling suicidal, their focus was more on the short-term rather than long-term goals, indicating the importance of encouraging respondents to include short-term goals when completing the AAT. While completing the Contemplating Suicide Section of the AAT, several participants stated that they had mainly focused on short-term goals and the immediate future when they last contemplated suicide. Consequently, they were less likely to focus on the kinds of long-term goals often listed by respondents when completing the AAT:

*“The big goals [when suicidal] were like to just do anything for my wellbeing, to shower and to eat and to leave the house, those were really big goals.” (Participant 2)*

*“Back then [when suicidal] I’d be like, okay, I – all I could think about is get through the day, get through the day, get through the week, get through the week.” (Participant 3)*

*“I wasn’t thinking strategically, I was just thinking of the immediate situation or the desire to just kind of stop things.” (Participant 9)*

Participants also expanded on the reasons for this focus on short-term goals, providing further support for the importance of assessing these short-term goals using the AAT. One participant explained that if they are feeling suicidal, it is helpful and necessary to focus on short-term goals (e.g., each step involved in taking a shower, such as shampooing their hair etc.). This is partly due to the achievability of these goals and partly because keeping a list of these daily goals reminds them of what tasks they need to complete while feeling distressed:

*“Short-term goals are really – I like them because it’s something that’s easily achievable. You know, you set your mind on something really, really small, it doesn’t feel too much pressure*… *I tend to forget what I’m doing a lot when I’m really kind of struggling, if it’s worse than normal. So I need that checklist [of daily short-term goals] to remind myself what I’m doing next.” (Participant 8)*

### 2.8 Stage 4: amendments to the AAT based on feedback from the cognitive interviews

Following analysis of the Stage 3 cognitive interviews, findings containing explicit feedback (e.g., direct feedback on problems with the phrasing of a particular item) and implicit feedback (e.g., descriptions of relevant experiences not already assessed by the AAT) informed AAT amendments in Stage 4. This was an iterative process involving the following: use of the AAT draft developed in Stage 2 for the first seven interviews; preliminary analysis of these interviews to identify potential improvements that should be made to the AAT; amendments to the AAT based on this preliminary analysis; interviews with the final two participants using the amended AAT draft (see Supplementary Data Sheets 4, 5 for the interviewer flowchart and answer booklet of this amended draft); further analysis of interview data; further refinements to the AAT based on feedback from these final two interviews.

Refinements after the first seven interviews and the final two involved: (i) examining cognitive interview data to identify problematic AAT items and participant feedback; (ii) research team discussions on amendments based on identified issues; (iii) implementing changes; and (iv) reviewing these amendments before finalizing the AAT version. All amendments made to the AAT in Stage 4 are described in the next section, and a more detailed list of these refinements is provided in Supplementary Table 1. Although refinements were not based on fixed empirical criteria, AAT items were deemed problematic (and therefore modified or excluded) if participants (i) found them difficult to comprehend; (ii) misunderstood them, leading to unexpected responses; (iii) described them as irrelevant to their experiences; or (iv) considered them unacceptable or distressing. Additionally, participant suggestions for adding relevant items or prompts/warnings (e.g., for suicide-related content) were incorporated, and difficulties the researcher encountered in administering certain items informed the refinements.

### 2.9 Stage 4: results

In response to the feedback on the AAT provided by participants within the cognitive interviews, a number of changes were made to refine and improve the AAT for use in future research (see Supplementary Data Sheets 6, 7 for the final version of the AAT following these amendments). Firstly, these included changes to the wording of AAT items themselves. The phrasing and length of questions throughout the AAT were reviewed and edited to improve their clarity. Additional options were added to some of the multiple-choice items, to reflect some participants’ experiences of mental imagery as both voluntary and involuntary, and of viewing it from both first and third-person perspective. In addition, an item was added to assess to what extent respondents have reached a particular goal (at the time of the interview) which they had when they last contemplated suicide. This will enable future researchers to tell if an individual’s goal has reduced in importance (between when they last contemplated suicide and the present) due to them having achieved it at the time of the interview, as opposed to other reasons for low ratings of goal importance. In addition, it ensures that failure to reach goals is recorded by the AAT.

Secondly, some of the instructions provided in the AAT of what guidance the interviewer should give respondents were edited. The explanation of what is meant by *goals*, which respondents are given by the interviewer prior to listing their goals, was edited to include more specific examples of a goal (e.g., “to go for a holiday in Majorca”), and to state that goals could be long-term or short-term. The additional examples include goals to avoid certain situations (e.g., “to avoid having an argument with a friend”). In addition, interviewer prompts were added to the AAT to explain some of the items assessing the ability to access one’s goals (e.g., “How able are you to bring this goal to mind?”), so that the interviewer gives examples such as the ability to visualize the goal or concentrate on it. This ensures that respondents in future research will fully understand the question. Lastly, guidance was added which instructs the interviewer to warn respondents when they are about to be asked questions about suicide, since they are less likely to be experienced as distressing if they are not a surprise and if the respondent is mentally prepared for them. The types of changes made to the AAT in Stage 4 and their justifications based on feedback from the Stage 3 cognitive interviews are summarized in Table 4. However, some Stage 3 themes (“The AAT as an acceptable and potentially helpful measure” and “Psychological processes regarding the potential impact of suicide captured by the AAT”) did not result in any amendments. This was because these themes highlighted strengths of the AAT and did not include feedback on any possible changes to the AAT.

**TABLE 4:** Summary of Stage 3 feedback and subsequent amendments to the Awareness Assessment Tool (AAT) in Stage 4.

Feedback	Relevant theme(s)	Subsequent amendment to the AAT
The phrasing and/or length of some items was experienced as confusing.	“Limitations of the AAT design”	Amendments to phrasing and length of AAT items to improve their clarity.
Participants’ experiences were not fully accounted for by some multiple-choice items (e.g., some participants experienced both voluntary and involuntary mental images).	“Limitations of the AAT design”	Additional options added to multiple-choice items on mental imagery.
Some goals were rated with low importance because they had already been reached. Some responses also indicated a potential role of failure to reach goals which should be accounted for.	“Limitations of the AAT design”; “The importance of accounting for failure to reach goals”	Items added to assess to what extent respondents have reached a particular goal.
Some participants only listed long-term goals due to limitations of the interviewer instructions. Feedback from participants indicated the importance of short-term goals and the necessity of ensuring that respondents list these goals where applicable.	“Limitations of the AAT design”; “The importance of assessing short-term goals”	Additional AAT interviewer instructions added to define goals using examples and state that short-term goals can be included.
Some items were experienced as ambiguous, leading to some unexpected responses, so further clarity is needed when explaining these items to respondents to ensure they are answered consistently.	“Limitations of the AAT design”	Addition of interviewer prompts to explain certain items to improve their clarity.
Some concerns were expressed about the potential for distress involved in introducing suicide-related questions without giving prior warning to respondents.	“Limitations of the AAT design”	Addition of interviewer instructions to warn respondents when they are about to be asked questions about suicide.

## 3 Discussion

The current study aimed to develop and refine the Awareness Assessment Tool (AAT), a new measure for assessing awareness of how suicide would impact on one’s personal goals (i.e., consequences of suicide). Firstly, this involved analysis of data from Macintyre et al. (2026a) on feedback regarding participants’ experiences of answering questions on suicide and personal goals (Stage 1) using Reflexive Thematic Analysis (TA). This analysis evaluated the potential of certain questions for inclusion in the AAT, highlighted potential difficulties in answering these kinds of questions, and informed subsequent AAT drafts (Stage 2). Secondly, cognitive interviews were conducted and analyzed using TA (Stage 3), with the aim of gaining feedback on participants’ experiences of completing the AAT. The cognitive interviews then informed improvements to subsequent AAT drafts (Stage 4). While the AAT has not yet undergone psychometric validation and therefore cannot be used as a clinical assessment tool in its current form, the findings suggest it shows promise pending further development.

Findings from Stage 1 and Stage 3 analyses indicated that questions on suicide, potential impact of suicide on participants’ personal goals, and mental imagery related to personal goals and suicide were experienced as safe and acceptable by participants. This is likely to be partially due to these questions being approached in a sensitive manner by the research team (e.g., informing participants in advance of the kinds of questions they would be asked and regularly “checking in” with participants during the interview). Indeed, it is essential to manage potential risks of participants experiencing distress when conducting suicide research (Andriessen, 2023). However, in both sets of interviews, a proportion of the participants experienced the questions on suicide and their personal goals as helpful, enabling them to make useful connections in their minds and compare their present and past experiences. This is consistent with findings that despite concerns that being asked about suicide and participating in suicide research could increase one’s risk of becoming suicidal, the opposite can often be the case (Andriessen, 2023; Bender et al., 2019; DeCou and Schumann, 2018; Poindexter et al., 2019). For example, a meta-analysis on the iatrogenic effect of conducting suicide screening assessments demonstrated that being screened for suicidality was not shown to increase participants’ risk of suicide (DeCou and Schumann, 2018). Furthermore, following an intensive research protocol involving suicide assessment and a task exposing participants to suicide-related imagery, Smith et al. (2010) found that most participants’ suicidal ideation decreased. Therefore, findings from the current study added to this body of research and provided some confirmation that the AAT is safe and acceptable for use in future research.

In addition to demonstrating the acceptability of being asked suicide-related questions, analysis of the data from Macintyre et al. (2026a) in Stage 1 highlighted the need to consider mood-congruent and mood-dependent memory when conducting research using the AAT. Mood-congruent memory refers to the influence of one’s mood on recall of emotional material, so that emotional material which is congruent with one’s current affective state is selectively retrieved and encoded (Faul and LaBar, 2023). Mood-dependent memory is a similar but separate phenomenon, occurring when neutral information is recalled more easily when it is retrieved while in the same mood as the mood in which it was encoded (Lewis and Critchley, 2003). Participants from Macintyre et al. (2026a) indicated that they were more likely to remember their experiences of contemplating/attempting suicide if they still experienced passive suicidal thoughts in the present. Conversely, participants who no longer experienced any suicidal thoughts found it more difficult to remember their experiences from contemplating/attempting suicide, due to their emotional state at that time being so different to their present emotional state. Some of these participants even described themselves as feeling like a different person from when they contemplated/attempted suicide. These findings indicate the potential for both mood-congruent and mood-dependent memory to influence recall when participants complete the AAT during future research, depending on to what extent they have recovered from experiencing suicidal thoughts. Given that one participant who experienced some difficulties in remembering their experiences had attempted suicide only a year prior to the interview, it is possible that participants’ recall is influenced more by mood-congruent memory than the length of time since their suicide attempt. If this is the case, it may be challenging to avoid this potential difficulty in recall of experiences in future research using the AAT.

Analysis of the data from the cognitive interviews in Stage 3 also highlighted further strengths and limitations of the AAT, including aspects of the experience of feeling suicidal which should be accounted for in an amended draft of the AAT. Firstly, the ability of the AAT to capture a variety of psychological processes that may be involved in awareness of consequences of suicide, highlighted by varying accounts from participants, was considered a strength. When responding to AAT questions, some participants described how suicide would completely interfere with a goal being reached and how this would deter them from ending their lives. This is consistent with recent findings that individuals are deterred from suicide when they have a clear awareness of how it could impact on their life goals (Macintyre et al., 2026a; Wynford-Thomas et al., 2026), and supports theoretical accounts that a greater awareness of how one’s goals could be impacted on by suicide is protective (Macintyre et al., 2021). Other participants described a variety of circumstances in which suicide would not interfere with the achievement of their goals, such as if the goal was so short-term and temporary (e.g., a GP appointment) that they could end their lives immediately afterward. From some of these participants’ perspectives, if the goal did not feel achievable, suicide would not interfere with the goal being reached as the goal could not be achieved anyway. It is possible that these kinds of accounts reflect a variety of thought processes by which individuals can “minimize” consequences of suicide (Tarrier et al., 2013), which were highlighted in a recent qualitative study (Macintyre et al., 2026a). Therefore, these findings, indicating a strength of the AAT in its ability to assess various psychological processes, provided support for the potential usefulness of the AAT items examining the perceived impact of suicide on respondents’ goals.

Secondly, accounts from participants indicated that failure to reach goals should be asked about by the AAT since some participants’ experiences of suicide were specifically linked to perceived failure to reach goals. Some participants described the stress they had experienced due to feeling unable to achieve a goal (e.g., to achieve good university grades), which had led to their suicidal thoughts. Moreover, some had considered suicide as a means of ending that stress and reducing the importance of reaching the goal, which they felt would help even though suicide would not enable them to actually reach the goal. This supports claims that some types of goal striving are associated with psychological distress (Gollwitzer and Oettingen, 2001) and that loss of control of one’s experiences can lead to distress (Carey et al., 2014). In addition, it supports findings that persistence in pursuing unattainable goals can lead to suicidal ideation and self-harm (O’Connor et al., 2012; Sandford et al., 2022). Furthermore, these findings provide some preliminary support for a recent hypothesis that individuals attempt suicide as a means of regaining control of their experiences (Macintyre et al., 2021) and are consistent with theoretical accounts of suicide as an alternative to life-oriented goals (Michel et al., 2017). Due to these findings, amendments were made to the AAT in Stage 4 so that respondents are asked to what extent they have reached a goal, enabling the role of failure to reach goals to be accounted for in future studies and/or assessments using the AAT. However, these findings also highlighted a potential limitation of the items examining the perceived impact of suicide on respondents’ goals, which aim to assess limited awareness of consequences of suicide. It is possible that while some responses to these items reflect limited awareness, others reflect a separate construct of using suicide as a means of regaining control of one’s experiences (Macintyre et al., 2021).

Lastly, analysis of the cognitive interviews highlighted that the AAT should include assessment of short-term goals in addition to longer-term goals. Findings indicated that some individuals focus almost exclusively on short-term goals when considering suicide, such as showering and eating. This aligns with research on memory specificity and future thinking. Studies show that memory specificity predicts the specificity of imagined future events, while overgeneral memory – which occurs in depressed and suicidal individuals – is associated with difficulty generating the concrete steps needed to achieve a goal (Williams et al., 1996, 2007). Accordingly, the exclusive focus on short-term goals observed in the current study may reflect a reduced capacity to envision the steps required to pursue longer-term goals during suicidal crises.

For some participants, this focus on achievable short-term goals while feeling suicidal may also be a strategy they employ to manage their suicidal thoughts. Qualitative findings from Macintyre et al. (2026a) revealed that some participants found it helpful to focus on short-term achievable goals in order to avoid acting on their suicidal thoughts and to continue functioning. Recent guidance on working with suicidal individuals advises practitioners to encourage the suicidal individual to focus on goals which feel achievable (Sandford et al., 2022), and it may be that short-term daily goals feel most achievable to individuals who are currently experiencing distress. Due to this importance of short-term goals during experiences of contemplating suicide, which was indicated by these findings, it was considered essential to include these goals in assessments using the AAT. Therefore, amendments were made to the AAT interviewer instructions in Stage 4 to ensure that participants would list short-term goals where applicable.

One strength of the current study was the fact that it developed and refined a novel method (the Awareness Assessment Tool; AAT) for assessing a recently hypothesized core psychological process which is posited to be involved in suicide (limited awareness of consequences of suicide) (Macintyre et al., 2021). This development process ensured the AAT was ready for use in a recent linked study, which further tested its acceptability and piloted its feasibility for use in larger quantitative studies (Macintyre et al., 2026b). Secondly, the sample contained some cultural diversity, although where possible, future research for evaluating the acceptability of the AAT should aim to gain feedback from individuals from a greater variety of cultures and backgrounds.

One limitation of the study was the fact that the Stage 1 and 3 samples were predominantly female. Although the AAT was acceptable to the Stage 3 sample, it may be less acceptable in predominantly male or differently gendered samples. Men face barriers such as self-stigma and are less likely to access mental health support (Sheikh et al., 2024; Üzümçeker, 2025), which may also shape how they experience AAT completion. Given that suicide is a leading cause of death in young men and rates are higher in men than women (Pitman et al., 2012; Struszczyk et al., 2019; White and Holmes, 2006), future research should evaluate AAT acceptability with larger male samples during further measure development. As a result of the gender imbalance in the present study, the findings primarily speak to acceptability in predominantly female samples, and additional work is required to establish acceptability among men.

In addition, the gender imbalance is likely to have influenced refinements to the AAT, since only limited feedback was gained from male participants. Similarly, AAT refinements and findings on acceptability were also limited by the small sample sizes – a larger sample may have highlighted a greater number of problematic AAT items or identified more acceptability issues. Given these limitations, future AAT validation work will recruit larger, more gender-balanced, and more diverse samples, including both general and clinical populations.

Lastly, recall bias likely occurred when participants completed the “Contemplating Suicide” section of the AAT, as it required them to recall potentially distressing experiences. Such bias is common in retrospective studies, particularly when emotional memories are involved (Ottenstein and Lischetzke, 2020; Talari and Goyal, 2020). As previously discussed, qualitative findings also suggested mood-dependent or mood-congruent memory effects, with some participants struggling to recall their experiences accurately. To minimize these issues in future AAT research, studies should avoid relying on retrospectively recalled data from the “Contemplating Suicide” section. Instead, where feasible (i.e., when mental health support can be provided), researchers should use only the “Present Time Experiences” section and adopt a longitudinal design with participants who remain at risk of suicide and may still have limited awareness. This consideration is important for future validation work, as recall bias may limit the validity of the “Contemplating Suicide” section, whereas the “Present Time Experiences” section may still demonstrate acceptable validity and reliability.

In terms of future research, we have described a number of refinements which have been made to the AAT following feedback from the cognitive interviews in order to improve its clarity, ease of use, and acceptability, so this new version of the AAT should be used in future studies. In addition, researchers should be aware of the potential for the following to influence participants’ responses when completing the AAT in future studies: mood-congruent or mood-dependent memory; perceptions of failure to reach goals; perceptions of suicide as a means of ending the distress arising from failure to achieve goals; a focus on short-term daily goals whilst individuals are suicidal.

Although the current study evaluated AAT item clarity using verbal probes and think-aloud techniques, further refinement is warranted. Future development could include additional cognitive interviews to assess and improve item clarity, as well as expert review in which item clarity and precision are rated using indices such as the Content Validity Index (CVI) or modified kappa (κ*) (Polit et al., 2007; Wynd et al., 2003).

Respondents’ emotional reactions were also assessed through questions addressing item acceptability and safety (e.g., “Was there anything about that question that made you feel less safe?”). An unpublished follow up study (Macintyre et al., 2026b)^3^, in which students completed the AAT alongside related measures, similarly evaluated emotional reactions through a brief feedback interview. As that study indicated a need for further refinement, additional cognitive interviews could use comparable topic guides to examine participants’ emotional responses after these revisions. These interviews could also explore goal-related biases through questions such as “What were you trying to convey with your answer?” or “Is there a “right” answer to this question?” complementing measures such as the Marlowe–Crowne Social Desirability Scale (MC-SDS) (Crowne and Marlowe, 1960).

In the unpublished follow up study (Macintyre et al., 2026b), scoring methods for seven of the AAT indices of awareness were developed. Scores from related items are combined to produce scores from 0 (indicating the least awareness possible) to 5 (the most awareness possible) on each of the indices of awareness. However, scoring methods for two indices of awareness which collect qualitative data (respondents’ ability to describe motives for their goals and respondents’ ability to describe steps to reach goals) have not yet been developed. It may be possible to score these remaining indices by quantifying specific versus vague steps and motives, in a method similar to the Means-End Problem Solving task (MEPS) (Platt and Spivack, 1975). Two or more researchers would independently code response specificity, and analysis would proceed once adequate inter-rater reliability is confirmed.

Given that the unpublished study (Macintyre et al., 2026b) aggregated multiple AAT items into each index of awareness, Exploratory and Confirmatory Factor Analyses could be conducted on these indices to evaluate the AAT’s psychometric properties. Each index of awareness would be expected to load onto a distinct factor. Convergent, divergent, and predictive validity could also be examined through associations between the indices and related measures. In addition, machine-learning techniques could be used to identify the most predictive algorithms for combining the indices of awareness. Machine learning has recently informed the development of other tools for suicide prevention (De Luca et al., 2024). Similarly, computational modeling – previously applied in PCT research to test hypotheses and model psychological change (Mansell and Huddy, 2020) – could be used to assess whether particular “low-awareness” profiles correspond to higher levels of suicide risk.

In future research and clinical practice (following validation), AAT administration is expected to require minimal training, as comprehensive interviewer instructions are provided. Scoring for the seven indices with an established system can be completed by following written instructions, requiring no additional training. Training may be needed only for the remaining two indices, to establish inter-rater reliability once their scoring system is developed.

After further development, the AAT may have clinical utility as a complement to comprehensive psychosocial assessments, which are recommended over reliance on risk assessment tools alone (National Institute for Health and Care Excellence., 2022). In addition, future research could use the AAT to examine links between awareness of consequences of suicide and factors such as childhood exposure to violence, aggression, abusive behavior, and risky behavior – each previously identified as a potential predictor of heightened suicide risk (Innamorati et al., 2011; Rossi et al., 2020). The AAT may also be useful in research and practice across high-stress contexts, including major societal or public health stressors. A recent review identified several risk factors for suicidal behavior during the COVID-19 pandemic among people with existing mental health difficulties, including psychological (e.g., hopelessness), interpersonal (e.g., isolation), and system-level factors (e.g., economic difficulties) (Barlattani et al., 2023). Because such factors may shape how individuals think about the future, their goals, and the potential impact of suicide, they could also influence awareness of consequences of suicide in similar high-stress settings. The AAT may therefore be valuable for detecting reduced awareness arising from these influences in such contexts.

### 3.1 Theoretical implications

The findings have several theoretical implications. Firstly, once validated, the AAT may offer added value beyond measures based on existing theoretical approaches by targeting a key construct posited to be involved in progressing from suicidal thoughts to action, i.e., limited awareness of consequences of suicide (Macintyre et al., 2021). A strength of the AAT highlighted in the themes from the cognitive interviews was its ability to capture multiple psychological processes that potentially reflect the construct of awareness of consequences of suicide, which is the AAT’s focus.

From a PCT perspective, this limited awareness would be considered a form of *arbitrary control* (Mansell, 2005; Morris and Mansell, 2018), a term referring to “attempts to make behavior conform to one set of goals without regard to other goals (and control systems) that may already be controlling that behavior” (Powers, 1973, p271). In the context of suicide, this reflects a narrow focus on suicide despite other goals that would be undermined by it (Macintyre et al., 2021).

From alternative theoretical perspectives, the construct of limited awareness could be viewed as a state factor that influences capability for suicide. Capability for suicide is a key predictor of attempts among people who experience suicidal ideation in the Interpersonal Theory of Suicide (IPTS; Joiner, 2005; Van Orden et al., 2010) and Three-step theory (3ST; Klonsky and May, 2015). Similarly, limited awareness would be considered a volitional moderator from the lens of the Integrated Motivational-Volitional model (IMV; O’Connor and Kirtley, 2018).

However, limited awareness does not map directly onto these frameworks, which conceptualize the transition from ideation to action as a linear process (Klonsky et al., 2018). By contrast, awareness of consequences of suicide is thought to fluctuate non-linearly (Macintyre et al., 2021), and may also relate to shifts in wish to live and wish to die (Oakey-Frost et al., 2023), as proposed in the Fluid Vulnerability Theory (FVT; Rudd, 2006). Therefore, use of the AAT to measure limited awareness may offer insights beyond research based on these theoretical approaches, due to its focus on a dynamic psychological process rather than static risk factors.

Because the AAT assesses only limited awareness and not the distress characteristic of suicidal crises, researchers and clinicians may wish to administer it alongside complementary measures. Tools for assessing constructs such as defeat, entrapment, and perceived burdensomeness – key predictors of suicidal ideation within frameworks such as the IMV and IPTS (Joiner, 2005; O’Connor and Kirtley, 2018; Van Orden et al., 2010) – could be included to provide a more comprehensive assessment of suicide risk.

Lastly, findings indicating that the AAT should be further developed to include items on failure to reach goals are consistent with PCT literature, which emphasizes the role of loss of control (i.e., the inability to reach one’s goals) in psychological distress (Carey, 2008; Powers, 1973). This loss of control and resulting distress may impact awareness of consequences of suicide, though further research is needed to investigate this potential link.

## 4 Conclusion

The current study aimed to develop, evaluate, and refine a new tool (the Awareness Assessment Tool; AAT) for assessing limited awareness of consequences of suicide, in a multi-stage iterative process of seeking and implementing feedback on the AAT. Reflexive Thematic Analysis of two sets of interview data (Stages 1 and 3) revealed themes indicating acceptability of the AAT, which has provided us with confidence that it is safe for use in future research. Analysis of data from cognitive interviews (Stage 3) also indicated a strength of the AAT in terms of its ability to assess various psychological processes involved in limited awareness of the potential impact of suicide. Lastly, a number of limitations of the AAT were identified from participants’ accounts and feedback on completing the AAT, enabling refinements to be made to the AAT (Stage 4). These findings have provided some information on the potential of the AAT for assessing limited awareness in suicidal individuals, although future research is necessary to further evaluate its acceptability and to pilot its feasibility for use in large quantitative studies.

## Data Availability

The data used in this article is not readily available to protect participant confidentiality and privacy. Further inquiries can be directed to the corresponding author.

## Footnotes

1 Macintyre, V. G., Pratt, D., Mansell, W., & Tai, S. J. (2026a). How do individuals describe their awareness of consequences from a time they contemplated or attempted suicide? Manuscript in Preparation.

2 Wynford-Thomas, N., Macintyre, V. G., & Tai, S. J. (2026). Assessing awareness of the consequences of suicide. Manuscript in Preparation.

3 Macintyre, V. G., Pratt, D., Mansell, W., & Tai, S. J. (2026b). The acceptability and feasibility of a tool for assessing awareness of consequences of suicide. Manuscript in Preparation
