# Supplementary material for: Development of a tool for assessing awareness of consequences of suicide
Source: Front Psychol. 2026 Feb 16;17:1736232. doi: 10.3389/fpsyg.2026.1736232 (PMC12950565; doi:10.3389/fpsyg.2026.1736232)
Supplement: Supplementary file 1 [file Data_Sheet_1.docx]

Supplementary Data Sheet 1: Interview topic guide for interviews conducted by Macintyre et al. (2026a)

- ***All writing in italics indicates information to be asked/said by the interviewer during the interview***

**Interview introduction**

- **Introduce self and aim of interview**
- *Firstly, thank you very much for volunteering to take part in this interview.*
- *I am a PhD student researching suicide at the University of Manchester.*
- *This interview has two aims. Firstly, I’d like to find out about your experiences of how* *aware you were of consequences of suicide when you thought about suicide or made a suicide attempt (if both apply to you).*
- *We hope that by gaining a better understanding of what leads someone to make an attempt, we might be able to develop a new clinical assessment tool.*
- *The second aim is to test how useful the interview questions are, because this interview is going to be developed into a more structured clinical interview later in my PhD project.*
- *In order to do this, I’d like your feedback on the interview so that your thoughts about the interview questions and structure can help shape the new clinical tool. Therefore, I would like to ask your opinion of the interview at the end.*
- *Any personal information (e.g. your name) will be removed when your interview is transcribed into text and it will not be possible to identify you from the data resulting from this or future studies. All information you give will be kept strictly confidential. However, if you told us any information that gave reason to believe you or someone else might be at risk of harming themselves or someone else, we would have to pass this information to the appropriate services, such as health care services, the police, or your GP.*
- *This interview should last up to an hour, depending on how much you want to say, and will be recorded using this audio device so that it can be analyzed later.*
- **Introduce types of questions, information we would like to gain and types of answers**
- *The interview will consist of a series of questions that aim to reveal your thoughts about suicide, your feelings when thinking about suicide and underlying reasons for suicide contemplation*
- *It is completely fine to answer ‘no’ decline to respond to specific questions*
- *There are no right answers, so do not worry about how you answer the questions. You might not even be sure of your answer if I ask you something you haven’t had an opportunity to think about before. We would just like an idea of the kind of thoughts you are having*
- **‘Think-aloud’ interviewing technique**
- *One of the interviewing techniques we will use today is called the ‘think-aloud’ technique*
- *When answering the questions that I ask you, I would like you to think aloud as much as possible, so that I can hear what you are thinking. In other words, rather than planning your answer and then speaking, I would like you to speak as you think*
- *Before we start the interview, I would like you to practise answering these types of questions, using an example unrelated to the main content of the interview, to help you answer questions in this way:*
- *Try to visualize the place where you live, and think about how many windows there are in that place. As you count up the windows, tell me what you are seeing and thinking about*
- **Remind them that they can stop at any point and encourage disclosure of any feelings of distress**
- *Before we start the interview, I would like to remind you that you can stop the interview completely at any point without giving a reason. You can also pause the interview at any point and take a break.*
- *I would also like to encourage you to tell me if at any point during the interview you are feeling distressed or upset*

**Demographics questions**

*Before I start the main interview, I would like to ask you a couple of demographics questions first about your previous experiences of suicide. These questions are completely optional and you do not have to answer them if you do not want to. If you do not want to answer either of these questions, please just let me know and we can move on to the next question or the main interview questions instead.*

1. *How long ago was your most recent suicide attempt (if applicable)?*
2. *How many times have you attempted suicide (if applicable)?*

**Main interview questions**

- Questions 1-3 will be repeated for each separate experience of contemplating/ attempting suicide (the number of separate experiences will vary depending on the participant – e.g. experiences of contemplating without making an attempt, experience of an attempt, and experiences of contemplating after the attempt):

1. *At the time you thought about attempting/attempted suicide, can you describe as much as you can remember about what came into your mind at that time?*
2. *When you have thought about attempting suicide/at the time that you attempted suicide, did you experience any images or verbal thoughts? (For all probes (a-i), if the participant answers ‘yes’ - ask ‘Can you describe what these experiences were?’)*
3. *Of a time you tried to harm yourself in the past*
4. *Of yourself planning/preparing to harm yourself or make a future suicide attempt*
5. *Of what might happen to you if you died*
6. *Of what might happen to other people if you died*
7. *Of things you were escaping from*
8. *Of another (non-suicide related) distressing event that happened to you (e.g. a trauma)*
9. *That made you feel safe or better*
10. *That were fleeting/unclear*
11. *Any other type*
12. *When you have felt suicidal/attempted suicide, did you think about things/goals that are important in your life? If so, what were these important things/goals?*
13. *How did thinking about these important things/goals affect your suicidal thoughts?*

*i) How did you feel when these important things/goals came into your mind*

*compared to times when you didn’t think of them?*

(If questions a) and ai) do not elicit the information we would like to gain, ask

question aii), aiii) and aiv))

*ii) When you thought about these important things/goals, did this make you*

*feel less suicidal?*

*iii) When you thought about these important things/goals, did it make you*

*feel more suicidal?*

*iv) What particular aspects of these important things/goals caused you to feel*

*less/more suicidal?*

1. *How prominent were the thoughts about the important things/goals?*

*i) How often did you think about these?*

(If the participant does not offer this information use the following prompts: *rarely, occasionally, often, very often*)

*ii) How strong were these?*

(If the participant does not offer this information use the following prompts: *weak, strong, very strong*)

1. *When thinking about these important things/goals, did you experience any images or verbal thoughts?*

*i) What were these?*

*ii) Can you describe the images?*

*iii) How do you feel when you describe these images? Is there anything else you notice?*

For each experience of contemplating/attempting suicide, after answering questions 1-3, participants will be asked the following questions on their recall of the experience:

1. *How well do you remember the experience you’ve just been describing?*
2. *How easy or hard to answer were the questions about this particular experience?*
3. *Are these things/goals still important to you now?*
4. *If they have changed – how?*
5. *Are there new things/goals that are important to you now that were not when you were feeling suicidal?*
6. *What is it that makes them important to you now?*
7. *What do you think about now when you think about suicide?*
8. *Are these thoughts the same or different from when you previously felt suicidal?*
9. *If different – how are they different?*
10. *What images come into your mind when you think about suicide now?*
11. *How do you feel when you are describing these images? Is there anything else you notice?*

**Question flow diagram**


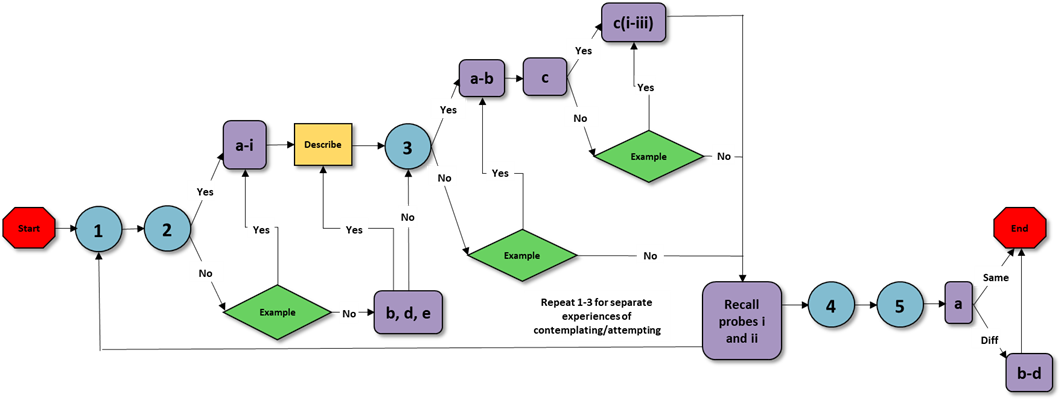


- Flow diagram illustrating how the interviews will be conducted to obtain maximum information from the participants. Arrows indicate the direction of question order. Text on arrowed lines indicates participant’s response – ‘same’ and ‘different’ refers to the participant’s response to probe question 5a, indicating whether their current thoughts about suicide are the same or different to when they previously felt suicidal. Main question numbers are shown in blue circles. Probe questions (small letters) are shown in purple rounded rectangles. When a participant is asked to describe their answer to probe question/s, this is shown in yellow rectangles. Provision of examples by interviewer (to ensure that the participant cannot provide any more information) is shown in green diamonds.

**Examples**

- Examples for certain questions will be given if a participant; asks for an example of an answer, does not understand a question, does not offer a response to a question or answers no to a question
- These examples need to provide a useful guide for the participant without leading them to specific answers

Specific examples

- Question 2 – example of experiencing images or visual thoughts:
- *‘For example, when you feel hungry you may imagine yourself eating something specific such as, experiencing the sweet taste of your favourite cake’*
- Probes 2b, d and e will be asked if the participant still answers ‘no’ to the main question 2 after example has been given
- This example can also be used for probe question 3c
- Question 3 – examples of a thing and a goal that could be important in someone’s life:
- *‘For example, for an older person, their grandchildren may be an important ‘thing’ in their life and having a successful retirement may be an important ‘goal’ in their life’*
- This example can be applied to later probe questions:
- Probe 3a – *thinking about their grandchildren may reduce this older person’s suicidal thoughts because they do not want to upset them. Whereas, thinking about the work needed to achieve a successful retirement may scare them and result in an increase in suicidal thoughts*

**After main interview**

(The main interview may need to be terminated before all questions are asked to ensure time for the participant to provide feedback about the interview)

- **Ask how they are and thank them again**
- *I finished asking you the interview questions*
- *Thank you for answering these questions*
- *How are you feeling?*
- **Feedback**
- *Would it be alright if I ask you a couple more questions to finish, to get your feedback of the interview?*
- *Can you tell me about your experience of the interview?*
- *How did you find the interview questions?*
- *Which questions felt more straight forward/difficult to answer?*
- *Were all or some of them difficult to answer? In what way did you find them straightforward/difficult?*
- *How did you find using the ‘think-aloud’ technique to answer the questions? How easy was it to do?*
- (If answer ‘not easy’) *Does that mean you would have preferred not to use the technique? Is there anything that could be done differently to make the questions easier to answer?*
- *In your opinion, were there; too many or the correct amount of questions asked?*
- (If ‘too many’) *Why?*
- (Only if examples were given*) Were the examples used helpful?*
- *Are there any ways in which the questions and/or examples could be improved?*
- **Feedback on acceptability/appropriateness of the interview:**
- *How appropriate did you feel the interview questions were?*
  - *(If ‘not appropriate’) What could be changed to make the questions more appropriate?*
- *Was there anything about the interview that you found less acceptable?*
  - *(If ‘yes’) What would / did enable the interview to become more acceptable to you?*
  - *(If ‘yes’) What could be changed to make the interview more acceptable?*
- *Was there anything about the interview that made you feel less safe?*
  - *(If ‘yes’) What could be changed to make you feel safer during the interview?*
- *Can you tell me about any concerns you had about answering the interview questions?*
- **Positive mood induction task**
- *I would like you to complete this positive mood induction task before you leave if that is alright*
- **Before they leave**
- Inform the participant that they will be emailed a copy of the ‘Support Services Contact Information Sheet’ immediately after the interview, and explain its use
