# Supplementary material for: Development of a tool for assessing awareness of consequences of suicide
Source: Front Psychol. 2026 Feb 16;17:1736232. doi: 10.3389/fpsyg.2026.1736232 (PMC12950565; doi:10.3389/fpsyg.2026.1736232)
Supplement: Supplementary file 2 [file Data_Sheet_2.pdf]

Supplementary Data Sheet 2: Interviewer flowchart for the initial Awareness Assessment Tool version used in the current study

Awareness Assessment Tool

Instructions for interviewers:

This Awareness Assessment Tool aims to assess the respondent's awareness of how their goals or values could be impacted on if they died by suicide. Since this awareness may fluctuate, the Awareness Assessment Tool consists of two sections. Section 1 consists of a series of questions relating to the respondent's awareness at the present moment, i.e. the time of completion of the Awareness Assessment Tool. Section 2 (from page 7 onwards) consists of a series of questions relating to the respondent's awareness at the time they most recently contemplated suicide.

Sections 1 and 2 can be completed in reverse order if required. Regardless of the order of completion, please provide a distraction break of 5 minutes between the completion of Section 1 and Section 2, to ensure that the responses from one section do not influence the responses in the other section. This distraction break should involve chatting about unrelated topics and/or be used as a comfort break.

When completing the Awareness Assessment Tool, the interviewer should read out each question to the respondent and use the suggested prompts whenever necessary, following the flow diagram of questions. The interviewer should fill in the answers for the respondent in the separate answer booklet. Each section of the answer booklet contains 8 pages which can be filled in for 8 separate goals, although these do not need to be filled in if the respondent is unable to think of 8 different goals.

When explaining the Awareness Assessment Tool to respondents, the interviewer should explain that depending on how the respondent feels on each occasion, the goals they list in question 2) of Section 1 might be the same as the goals listed in question 3) of Section 2, or they might be different, and that both are fine. The interviewer should explain that the reason the respondent is being asked such similar questions in different sections of the assessment is that this will vary between different people, and whether the goals listed are different for each section depends on the individual completing the assessment.

All instructions for the interviewer are provided in italics. Instructions for scoring the Awareness Assessment Tool are provided separately.

Section 1 – Awareness of goals at the present time

1) Current mood state: What is your current mood at the present moment?

The interviewer should read out the words from the answer booklet which describe different feelings and emotions, or show them to the respondent. The interviewer should then mark the appropriate answer in the space next to each word, once the respondent has indicated to what extent they feel that way right now, that is, at the present moment.

2) Goals: Can you list one or more goals that are important to you?

By “goals” we mean future experiences that people generally try to achieve or accomplish (for example, have a successful career, get a promotion at work, be a good parent) as well as experiences that people generally try to avoid (for example, someone could have a goal to feel less lonely or to stop feeling distressed). Goals can be related to something you are already doing, and you want to keep doing, or even something realistic that you would like to achieve in future. Also, goals can be either very specific (such as something that you would like to do, like going for a holiday in a specific place) or they can be more general (like getting on with your family members or being a nice person).

If they list 1-3 goals, move on to next question

If they cannot list one or more goals, use the following prompts

“What is living about for you?”

“Can you give an example of what you do on an everyday basis?”

“Can you picture things you would be doing if things were going right for you?”

“If you could wave a magic wand and not have suicidal thoughts for a day, what would that day look like?”

“What is your future self doing if your problems are resolved?”

“Are there any specific life experiences you would like to have if things were different?

If no goals are listed, use more specific examples:

“How important is your family to you?”

If they list 1-3 goals, move on to next question

If they cannot list one or more goals

*Skip to question **3 a)***

2 a) Importance of goals: *For each goal, ask the respondent to answer the following question:*

How important is it for you to accomplish this goal on a scale from 0 to 10?

2 b) Underlying higher-level motives for each goal: *For each goal, ask the respondent to answer the following prompt questions until they describe why the goal is important to them:*

"Why is [goal] important to you?"

"Why does this help?"

"Why do you like [goal]?

"What would be good about achieving [goal]?"

"If you achieved that goal, what would that mean to you?"

Move onto next question once they have been prompted to explain why each goal is important to them, even if they are unable to list any reasons for wanting to achieve any of the goals

2 c) Ability to access each goal: *For each goal, ask the respondent to answer the following questions:*

i) How able are you to bring this goal to mind? (0 = not at all, 5 = extremely able)
ii) How often do you think about this? (more than once a day, once a day, once a week, once a month, once every few months, never)

2 d) Means of achieving each goal: *For each goal, ask the respondent to briefly describe how they would achieve this goal using the following prompts:*

"Thinking about your current situation right now, how would you achieve this?"

"What would need to happen for this to be possible?"

If they cannot describe any means of achieving any of the goals they have listed

If some means of achieving some of the goals are

Skip to question ***2 f)***

2 e) Identifying external barriers to achieving each goal: *For each goal, ask the respondent the following questions:*

i) What gets in the way of you achieving this goal?
ii) What would happen if you tried to achieve this goal?
iii) What choices do you feel you have at the moment in terms of achieving this goal?

2 f) Impact of suicide on goals: *For each goal, ask the respondent to answer the following questions:*

i) If you died by suicide, how much would it interfere with the achievement of this goal? (0 = not at all, 10 = very much so)
ii) If you died by suicide, could you still achieve this goal? (Yes/No)
iii) If you died by suicide, how much would it help with achieving this goal? (0 = not at all, 10 = very much so)

2 g) Goal-related mental imagery: *For each goal, ask the respondent to answer the following question:*

Do any mental images come into your mind when you think of this goal? (Yes/No)

Yes

No

Skip to question ***3 a)***

2 h) Imagery description: Can you describe the images?

Instructions for interviewer: If any prompts are needed, provide the example of someone who wants to eat a cake might have images in their mind of what the icing would look like and how it would taste.

2 i) Imagery details: *For each goal, as the respondent to answer the following questions:*

i) How vivid are the images? (0 = not vivid at all, 5 = very vivid)
ii) Do you experience the images as though it is through your own eyes (first person perspective) or as though you are watching yourself in the image (third person perspective)?
iii) Are the images voluntary (i.e. do you deliberately imagine them) or involuntary (i.e. do they just come into your mind spontaneously)?

If no goals were listed in question 2), move to question 3).
Otherwise, skip question 3) and move on to the next section of

3 a) Other imagery: Are you experiencing any other mental images right now, at this moment?

Yes

No

End this section of the assessment

3 b) Imagery description: Can you describe the images?

Instructions for interviewer: If any prompts are needed, provide the example of someone who wants to eat a cake might have images in their mind of what the icing would look like and how it would taste.

3 c) Imagery details: *For each image, ask the respondent to answer the following questions:*

i) How vivid is the image? (0 = not vivid at all, 5 = extremely)
ii) Are you experiencing the images as though it is through your own eyes (first person perspective) or as though you are watching yourself in the image (third person perspective)?
iii) Are the images voluntary (i.e. do you deliberately imagine them) or involuntary (i.e. do they just come into your mind spontaneously)?
iv) To what extent are you trying to keep the images out of your mind? (0 = not at all, 5 = every time I experience the image)

3 d) Goals which come to mind when focusing on imagery: *For each image, ask the respondent to answer the following question:*

As you focus on this image, do any goals come to mind that you have, which did not come into your mind during the previous questions about goals?

If yes, repeat questions 2a) – 2f) for each goal listed while responding to this question

If no, move onto Section 2 if applicable

Section 2 – Awareness of goals at the time they most recently contemplated suicide

Instructions for interviewer: This section follows a similar structure to Section 1, but should focus on the specific occasion when the respondent most recently contemplated suicide.

1) Mood state: How would you describe your mood at the time you most recently contemplated suicide?

The interviewer should read out the words from the answer booklet which describe different feelings and emotions, or show them to the respondent. The interviewer should then mark the appropriate answer in the space next to each word, once the respondent has indicated to what extent they felt that way at the time they most recently contemplated suicide.

↓

2) Ability to access each goal listed in Section 1: *For each goal which was previously listed in Section 1, ask the respondent to answer the following questions:*

i) How easy did you find it to bring this goal to mind at the time you most recently contemplated suicide? (0 = not at all, 5 = very much so)

ii) How often did you think about the goal at that time? (more than once a day, once a day, once a week, once a month, once every few months, never)

iii) To what extent did it influence your decisions at that time? (0 = not at all, 5 = very much so)

↓

3) Goals: Can you list one or more goals that are important to you, which came into your mind when you most recently contemplated suicide?

By "goals" we mean future experiences that people generally try to achieve or accomplish (for example, have a successful career, get a promotion at work, be a good parent) as well as experiences that people generally try to avoid (for example, someone could have a goal to feel less lonely or to stop feeling distressed). Goals can be related to something you are already doing, and you want to keep doing, or even something realistic that you would like to achieve in future. Also, goals can be either very specific (such as something that you would like to do, like going for a holiday in a specific place) or they can be more general (like getting on with your family members or being a nice person).

If they list 1-3 goals, move on to next question

If they cannot list one or more goals, use the following prompts

"What is living about for you?"

"Can you give an example of what you do on an everyday basis?"

"Can you picture things you would be doing if things were going right for you?"

"If you could wave a magic wand and not have suicidal thoughts for a day, what would that day look like?"

"What is your future self doing if your problems are resolved?"

"Are there any specific life experiences you would like to have if things were different?

If no goals are listed, use more specific examples:

"How important is your family to you?"

"How important is your career to you?"

If they list 1-3 goals, move on to next question

If they cannot list one or more goals

Skip to question ***4 a)***

3 a) Importance of goals: *For each goal, ask the respondent to answer the following question:*

How important is was for you to accomplish this goal on a scale from 0 to 10, at the time you most recently contemplated suicide?

3 b) Underlying higher-level motives for each goal: *For each goal, ask the respondent to answer the following prompt questions until they describe why the goal is important to them. For any goals which are the same as the goals listed in Section 1, move onto the next goal if applicable. If this is not applicable, move to question **3c)**.*

"Why is [goal] important to you?"

"Why does this help?"

"Why do you like [goal]?

"What would be good about achieving [goal]?"

Move onto next question once they have been prompted to explain why each goal is important to them, even if they are unable to list any reasons for wanting to achieve any of the goals

3 c) Ability to access each goal: *For each goal, ask the respondent to answer the following questions:*

i) How easy did you find it to bring this goal to mind at the time you most recently contemplated suicide? (0 = not at all, 5 = very much so)

ii) How often did you think about the goal at that time? (more than once a day, once a day, once a week, once a month, once every few months, never)

3 d) Means of achieving each goal: *For each goal, ask the respondent to briefly describe how they would achieve this goal using the following prompts. For any goals which are the same as the goals listed in Section 1, move onto the next goal if applicable. If this is not applicable, move to question* ***3e)****.*

"Thinking about your current situation right now, how would you achieve this?"

"What would need to happen for this to be possible?"

If they cannot describe any means of achieving any of the goals they have listed → 3 e)

If some means of achieving some of the goals are → *Skip to question* ***3 f)***

3 e) Identifying external barriers to achieving each goal: *For each goal, ask the respondent the following questions:*

i) What got in the way of you achieving this goal at the time you most recently contemplated suicide?
ii) What would have happened if you had tried to achieve this goal around the time that you most recently contemplated suicide?
iii) What choices did you feel you had in terms of achieving this goal, at the time you most recently contemplated suicide?

3 f) Impact of suicide on goals: *For each goal, ask the respondent to answer the following questions:*

i) If you died by suicide, how much would it interfere with the achievement of this goal? (0 = not at all, 5 = very much so)
ii) If you died by suicide, could you still achieve this goal? (Yes/No)
iii) If you died by suicide, how much would it help with achieving this goal? (0 = not at all, 5 = very much so)

3 g) Goal-related mental imagery: *For each goal, ask the respondent to answer the following question:*

Did any mental images come into your mind when you think of this goal when you most recently contemplated suicide? (Yes/No)

Yes

No

Skip to question ***4 a)***

3 h) Imagery description: Can you describe the images?

Instructions for interviewer: If any prompts are needed, provide the example of someone who wants to eat a cake might have images in their mind of what the icing would look like and how it would taste.

3 i) Imagery details: *For each goal, as the respondent to answer the following questions:*

i) How vivid are the images? (0 = not vivid at all, 5 = very vivid)
ii) Do you experience the images as though it is through your own eyes (first person perspective) or as though you are watching yourself in the image (third person perspective)?
iii) Are the images voluntary (i.e. do you deliberately imagine them) or involuntary (i.e. do they just come into your mind spontaneously)?

If the goal answer booklet for question 3) is not full, move on to question 4). Otherwise, skip question 4).

4 a) Other imagery: Did any other mental images come into your mind when you most recently contemplated suicide, which are **not** related to these goals?

Yes

No

End this section of the assessment

4 b) Imagery description: Can you describe the images?

Instructions for interviewer: If any prompts are needed, provide the example of someone who wants to eat a cake might have images in their mind of what the icing would look like and how it would taste.

4 c) Imagery details: *For each image, ask the respondent to answer the following questions:*

i) How vivid was the image? (0 = not vivid at all, 5 = extremely)
ii) Did you experience the images as though it is through your own eyes (first person perspective) or as though you are watching yourself in the image (third person perspective)?
iii) Were the images voluntary (i.e. do you deliberately imagine them) or involuntary (i.e. do they just come into your mind spontaneously)?
iv) To what extent did you try to keep the images out of your mind? (0 = not at all, 5 = every time I experienced the image)

4 d) Goals which come to mind when focusing on imagery: *For each image, ask the respondent to answer the following question:*

As you focus on this image now, do any goals come to mind that you have, which did not come into your mind during the previous questions about goals?

Yes

Repeat questions 3a) – 3f) for each goal listed in the response to this question

No

End this section of the assessment
