# Supplementary material for: Development of a tool for assessing awareness of consequences of suicide
Source: Front Psychol. 2026 Feb 16;17:1736232. doi: 10.3389/fpsyg.2026.1736232 (PMC12950565; doi:10.3389/fpsyg.2026.1736232)
Supplement: Supplementary file 3 [file Data_Sheet_3.pdf]

Supplementary Data Sheet 3: Answer booklet for the initial Awareness Assessment Tool version used in the current study

Awareness Assessment Tool answer booklet

Section 1 – Awareness of goals at the present time

1) Current mood state

Indicate the extent you feel the following emotions right now, that is, at the present moment	Very slightly or not at all - 1	A little - 2	Moderately - 3	Quite a bit - 4	Extremely - 5
Interested					
Distressed					
Excited					
Upset					
Strong					
Guilty					
Scared					
Hostile					
Enthusiastic					
Proud					

Irritable					
Alert					
Ashamed					
Inspired					
Nervous					
Determined					
Attentive					
Jittery					
Active					
Afraid					

2) Goals

Goal	Importance (0-10)
1)	

2 b) Underlying higher-level motives for each goal:

2 c) Ability to access each goal:

i) ☐

ii)

- ☐ More than once a day
- ☐ Once a day
- ☐ Once a week
- ☐ Once a month
- ☐ Once every few months
- ☐ Never

iii)

2 d) Means of achieving this goal (subgoals):

2 e) Identifying external barriers to achieving each goal:

i)

ii)

iii)

2 f) Impact of suicide on goals:

i)

ii)

iii)

2 g) Goal-related mental imagery:

2 h) Imagery description:

2 i) Imagery details:

i)

ii)

iii)

iv)

**Goal**	**Importance (0-10)**
**2)**	

2 b) Underlying higher-level motives for each goal:

2 c) Ability to access each goal:

i) ☐

ii)

- ☐ More than once a day
- ☐ Once a day
- ☐ Once a week
- ☐ Once a month
- ☐ Once every few months
- ☐ Never

iii) ☐

2 d) Means of achieving this goal (subgoals):

2 e) Identifying external barriers to achieving each goal:

i)

ii)

iii)

2 f) Impact of suicide on goals:

i)

ii)

iii)

2 g) Goal-related mental imagery:

2 h) Imagery description:

2 i) Imagery details:

i)

ii)

iii)

iv)

Goal	Importance (0-10)
3)	

2 b) Underlying higher-level motives for each goal:

2 c) Ability to access each goal:

i) ☐

ii)

- ☐ More than once a day
- ☐ Once a day
- ☐ Once a week
- ☐ Once a month
- ☐ Once every few months
- ☐ Never

iii) ☐

2 d) Means of achieving this goal (subgoals):

2 e) Identifying external barriers to achieving each goal:

i)

ii)

iii)

2 f) Impact of suicide on goals:

i)

ii)

iii)

2 g) Goal-related mental imagery:

2 h) Imagery description:

2 i) Imagery details:

i)

ii)

iii)

iv)

**Goal**	**Importance (0-10)**
**4)**	

2 b) Underlying higher-level motives for each goal:

2 c) Ability to access each goal:

i) ☐

ii)

- ☐ More than once a day
- ☐ Once a day
- ☐ Once a week
- ☐ Once a month
- ☐ Once every few months
- ☐ Never

iii) ☐

2 d) Means of achieving this goal (subgoals):

2 e) Identifying external barriers to achieving each goal:

i)

ii)

iii)

2 f) Impact of suicide on goals:

i)

ii)

iii)

2 g) Goal-related mental imagery:

2 h) Imagery description:

2 i) Imagery details:

i)

ii)

iii)

iv)

**Goal**	**Importance (0-10)**
**5)**	

2 b) Underlying higher-level motives for each goal:

2 c) Ability to access each goal:

i) ☐

ii)

- ☐ More than once a day
- ☐ Once a day
- ☐ Once a week
- ☐ Once a month
- ☐ Once every few months
- ☐ Never

iii) ☐

2 d) Means of achieving this goal (subgoals):

2 e) Identifying external barriers to achieving each goal:

i)

ii)

iii)

2 f) Impact of suicide on goals:

i)

ii)

iii)

2 g) Goal-related mental imagery:

2 h) Imagery description:

2 i) Imagery details:

i)

ii)

iii)

iv)

**Goal**	**Importance (0-10)**
**6)**	

2 b) Underlying higher-level motives for each goal:

2 c) Ability to access each goal:

i) ☐

ii)

- ☐ More than once a day
- ☐ Once a day
- ☐ Once a week
- ☐ Once a month
- ☐ Once every few months
- ☐ Never

iii) ☐

2 d) Means of achieving this goal (subgoals):

2 e) Identifying external barriers to achieving each goal:

i)

ii)

iii)

2 f) Impact of suicide on goals:

i)

ii)

iii)

2 g) Goal-related mental imagery:

2 h) Imagery description:

2 i) Imagery details:

i)

ii)

iii)

iv)

Goal	Importance (0-10)
7)	

2 b) Underlying higher-level motives for each goal:

2 c) Ability to access each goal:

i) ☐

ii)

- ☐ More than once a day
- ☐ Once a day
- ☐ Once a week
- ☐ Once a month
- ☐ Once every few months
- ☐ Never

iii) ☐

2 d) Means of achieving this goal (subgoals):

2 e) Identifying external barriers to achieving each goal:

i)

ii)

iii)

2 f) Impact of suicide on goals:

i)

ii)

iii)

2 g) Goal-related mental imagery:

2 h) Imagery description:

2 i) Imagery details:

i)

ii)

iii)

iv)

**Goal**	**Importance (0-10)**
**8)**	

2 b) Underlying higher-level motives for each goal:

2 c) Ability to access each goal:

i) ☐

ii)

- ☐ More than once a day
- ☐ Once a day
- ☐ Once a week
- ☐ Once a month
- ☐ Once every few months
- ☐ Never

iii) ☐

2 d) Means of achieving this goal (subgoals):

2 e) Identifying external barriers to achieving each goal:

i)

ii)

iii)

2 f) Impact of suicide on goals:

i)

ii)

iii)

2 g) Goal-related mental imagery:

2 h) Imagery description:

2 i) Imagery details:

i)

ii)

iii)

iv)

3 a) Other mental imagery:

3 b) Imagery description:

3 c) Imagery details:

First image:

i)

ii)

iii)

iv)

Second image:

i)

ii)

iii)

iv)

Third image:

**i)**	
**ii)**	
**iii)**	
**iv)**	

Fourth image:

**i)**	
**ii)**	
**iii)**	
**iv)**	

Fifth image:

**i)**	
**ii)**	
**iii)**	
**iv)**	

Section 2 – Awareness of goals at the time they most recently contemplated suicide

1) Mood state

Indicate the extent you feel the following emotions right now, that is, at the present moment	Very slightly or not at all - 1	A little - 2	Moderately - 3	Quite a bit - 4	Extremely - 5
Interested					
Distressed					
Excited					
Upset					
Strong					
Guilty					
Scared					
Hostile					
Enthusiastic					
Proud					
Irritable					
Alert					
Ashamed					
Inspired					
Nervous					

Determined					
Attentive					
Jittery					
Active					
Afraid					

2) Ability to access each goal listed in Section 1 during contemplation

Goal 1:

i) ☐

ii)

☐ More than once a day
☐ Once a day
☐ Once a week
☐ Once a month
☐ Once every few months
☐ Never

iii) ☐

Goal 2:

i) ☐

ii)

☐ More than once a day
☐ Once a day
☐ Once a week

☐ Once a month

☐ Once every few months

☐ Never

iii) ☐

Goal 3:

i) ☐

ii)

☐ More than once a day

☐ Once a day

☐ Once a week

☐ Once a month

☐ Once every few months

☐ Never

iii) ☐

Goal 4:

i) ☐

ii)

☐ More than once a day

☐ Once a day

☐ Once a week

☐ Once a month

☐ Once every few months

☐ Never

iii) ☐

Goal 5:

i) ☐

ii)

☐ More than once a day

☐ Once a day

☐ Once a week

☐ Once a month

☐ Once every few months

☐ Never

iii) ☐

Goal 6:

i) ☐

ii)

☐ More than once a day

☐ Once a day

☐ Once a week

☐ Once a month

☐ Once every few months

☐ Never

iii) ☐

Goal 7:

i) ☐

ii)

- ☐ More than once a day
- ☐ Once a day
- ☐ Once a week
- ☐ Once a month
- ☐ Once every few months
- ☐ Never

iii) ☐

Goal 8:

i) ☐

ii)

- ☐ More than once a day
- ☐ Once a day
- ☐ Once a week
- ☐ Once a month
- ☐ Once every few months
- ☐ Never

iii)

3) Goals

Goal	Importance (0-10)
1)	

3 b) Underlying higher-level motives for each goal:

3 c) Ability to access each goal:

i) ☐

ii)

- ☐ More than once a day
- ☐ Once a day
- ☐ Once a week
- ☐ Once a month
- ☐ Once every few months
- ☐ Never

iii)

3 d) Means of achieving this goal (subgoals):

3 e) Identifying external barriers to achieving each goal:

i)

ii)

iii)

3 f) Impact of suicide on goals:

i)

ii)

iii)

3 g) Goal-related mental imagery:

3 h) Imagery description:

3 i) Imagery details:

i)

ii)

iii)

iv)

**Goal**	**Importance (0-10)**
**2)**	

3 b) Underlying higher-level motives for each goal:

3 c) Ability to access each goal:

i) ☐

ii)

- ☐ More than once a day
- ☐ Once a day
- ☐ Once a week
- ☐ Once a month
- ☐ Once every few months
- ☐ Never

iii) ☐

3 d) Means of achieving this goal (subgoals):

3 e) Identifying external barriers to achieving each goal:

i)

ii)

iii)

3 f) Impact of suicide on goals:

i)

ii)

iii)

3 g) Goal-related mental imagery:

3 h) Imagery description:

3 i) Imagery details:

i)

ii)

iii)

iv)

**Goal**	**Importance (0-10)**
**3)**	

3 b) Underlying higher-level motives for each goal:

3 c) Ability to access each goal:

i) ☐

ii)

- ☐ More than once a day
- ☐ Once a day
- ☐ Once a week
- ☐ Once a month
- ☐ Once every few months
- ☐ Never

iii) ☐

3 d) Means of achieving this goal (subgoals):

3 e) Identifying external barriers to achieving each goal:

i)

ii)

iii)

3 f) Impact of suicide on goals:

i)

ii)

iii)

3 g) Goal-related mental imagery:

3 h) Imagery description:

3 i) Imagery details:

i)

ii)

iii)

iv)

**Goal**	**Importance (0-10)**
**4)**	

3 b) Underlying higher-level motives for each goal:

3 c) Ability to access each goal:

i) ☐

ii)

- ☐ More than once a day
- ☐ Once a day
- ☐ Once a week
- ☐ Once a month
- ☐ Once every few months
- ☐ Never

iii) ☐

3 d) Means of achieving this goal (subgoals):

3 e) Identifying external barriers to achieving each goal:

i)

ii)

iii)

3 f) Impact of suicide on goals:

i)

ii)

iii)

3 g) Goal-related mental imagery:

3 h) Imagery description:

3 i) Imagery details:

i)

ii)

iii)

iv)

**Goal**	**Importance (0-10)**
**5)**	

3 b) Underlying higher-level motives for each goal:

3 c) Ability to access each goal:

i) ☐

ii)

- ☐ More than once a day
- ☐ Once a day
- ☐ Once a week
- ☐ Once a month
- ☐ Once every few months
- ☐ Never

iii) ☐

3 d) Means of achieving this goal (subgoals):

3 e) Identifying external barriers to achieving each goal:

i)

ii)

iii)

3 f) Impact of suicide on goals:

i)

ii)

iii)

3 g) Goal-related mental imagery:

3 h) Imagery description:

3 i) Imagery details:

i)

ii)

iii)

iv)

Goal	Importance (0-10)
6)	

3 b) Underlying higher-level motives for each goal:

3 c) Ability to access each goal:

i) ☐

ii)

- ☐ More than once a day
- ☐ Once a day
- ☐ Once a week
- ☐ Once a month
- ☐ Once every few months
- ☐ Never

iii) ☐

3 d) Means of achieving this goal (subgoals):

3 e) Identifying external barriers to achieving each goal:

i)

ii)

iii)

3 f) Impact of suicide on goals:

i)

ii)

iii)

3 g) Goal-related mental imagery:

3 h) Imagery description:

3 i) Imagery details:

i)

ii)

iii)

iv)

**Goal**	**Importance (0-10)**
**7)**	

3 b) Underlying higher-level motives for each goal:

3 c) Ability to access each goal:

i) ☐

ii)

☐ More than once a day

☐ Once a day

☐ Once a week

☐ Once a month

☐ Once every few months

☐ Never

iii) ☐

3 d) Means of achieving this goal (subgoals):

3 e) Identifying external barriers to achieving each goal:

i)

ii)

iii)

3 f) Impact of suicide on goals:

i)

ii)

iii)

3 g) Goal-related mental imagery:

3 h) Imagery description:

3 i) Imagery details:

i)

ii)

iii)

iv)

Goal	Importance (0-10)
8)	

3 b) Underlying higher-level motives for each goal:

3 c) Ability to access each goal:

i) ☐

ii)

- ☐ More than once a day
- ☐ Once a day
- ☐ Once a week
- ☐ Once a month
- ☐ Once every few months
- ☐ Never

iii) ☐

3 d) Means of achieving this goal (subgoals):

3 e) Identifying external barriers to achieving each goal:

i)

ii)

iii)

3 f) Impact of suicide on goals:

i)

ii)

iii)

3 g) Goal-related mental imagery:

3 h) Imagery description:

3 i) Imagery details:

i)

ii)

iii)

iv)

4 a) Other mental imagery:

4 b) Imagery description:

4 c) Imagery details:

First image:

i)

ii)

iii)

iv)

Second image:

i)

ii)

iii)

iv)

Third image:

i)	☐
ii)	☐
iii)	☐
iv)	☐

Fourth image:

i)	☐
ii)	☐
iii)	☐
iv)	☐

Fifth image:

i)	☐
ii)	☐
iii)	☐
iv)	☐
