# Supplementary material for: Development of a tool for assessing awareness of consequences of suicide
Source: Front Psychol. 2026 Feb 16;17:1736232. doi: 10.3389/fpsyg.2026.1736232 (PMC12950565; doi:10.3389/fpsyg.2026.1736232)
Supplement: Supplementary file 6 [file Data_Sheet_6.pdf]

Supplementary Data Sheet 6: Flowchart and interviewer instructions for the final version of the Awareness Assessment Tool

Awareness Assessment Tool

Instructions for interviewers:

This Awareness Assessment Tool aims to assess the respondent's awareness of how their goals or values could be impacted on if they died by suicide. Since this awareness may fluctuate, the Awareness Assessment Tool consists of two sections. Section 1 consists of a series of questions relating to the respondent's awareness at the present moment, i.e. the time of completion of the Awareness Assessment Tool. Section 2 (from page 12 onwards) consists of a series of questions relating to the respondent's awareness at the time they most recently contemplated suicide.

Sections 1 and 2 can be completed in reverse order if required. Regardless of the order of completion, please provide a distraction break of 5 minutes between the completion of Section 1 and Section 2, to ensure that the responses from one section do not influence the responses in the other section. This distraction break should involve chatting about unrelated topics and/or be used as a comfort break. Before beginning Section 2, please warn the respondent that it will consist of questions about when they most recently contemplated suicide, and encourage them to say if they feel uncomfortable and need to take extra breaks during this section.

When completing the Awareness Assessment Tool, the interviewer should read out each question to the respondent and use the suggested prompts whenever necessary, following the flow diagram of questions. The respondent should answer verbally and the interviewer should fill in the answers for the respondent in the separate answer booklet. Each section of the answer booklet contains 3 sets of pages which can be filled in for 3 separate goals, although these do not need to be filled in if the respondent is unable to think of 3 different goals.

In both sections, when following the flow diagram, for questions involving more than one goal, please focus on one goal at a time and complete all questions for each goal before moving onto the next goal. E.g. complete questions 1 a) – 1 j) for the first goal before moving onto the second goal, and then complete questions 1 a) – 1 j) for the second goal, and so on. When asking each question, please make it clear to the participant which goal you are asking about.

When explaining the Awareness Assessment Tool to respondents, the interviewer should explain that there are two separate sections (Sections 1 and 2) and that depending on how the respondent feels on each occasion, the goals they list in question 1) of Section 1 might be the same as the goals listed in question 2) of Section 2, or they might be different, and that both are fine. The interviewer should explain that the reason the respondent is being asked such similar questions in different sections of the assessment is that this will vary between different people, and whether the goals listed are different for each section depends on the individual completing the assessment.

All instructions for the interviewer are provided in italics. Instructions for scoring the Awareness Assessment Tool are provided separately.

Current mood state: What is your current mood at the present moment?

The interviewer should read out the words from the answer booklet which describe different feelings and emotions, or show them to the respondent. The interviewer should then mark the appropriate answer in the space next to each word, once the respondent has indicated to what extent they feel that way right now, that is, at the present moment.

Section 1 – Awareness of goals at the present time

1) Goals: Can you list one or more goals that are important to you?

In this assessment, the term "goals" has a broader definition than the one you might normally use. By "goals" we mean future experiences that people generally try to achieve or accomplish (for example, have a successful career, get a promotion at work, be a good parent) as well as experiences that people generally try to avoid (for example, someone could have a goal to feel less lonely or to stop feeling distressed). Goals can be related to something you are already doing, and you want to keep doing (for example, you might have a good relationship with your family members that you want to maintain, or you might want to continue doing well at your job or university work). Goals can be either very specific, such as something that you would like to do or achieve (e.g. go for a holiday in Majorca or pass your driving test) or something specific that you would want to avoid (e.g. being late for an appointment or having an argument with a friend), and they can be either long-term or short-term. Goals can also be more general, such as a goal to help people or be a nice person, or a goal to avoid being a bad person.

If the participant lists more than 3 goals, ask the following question, and then write the 3 goals they answer with in the goals section of the answer booklet:

Which three of these goals are most relevant in your life or do you spend the most time focusing on?

If they list 1-3 goals, move on to next question

If they cannot list one or more goals, use the following prompts

"What is living about for you?"
"Can you give an example of what you do on an everyday basis?"
"Can you picture things you would be doing if things were going right for you?"
"If you could wave a magic wand and not have suicidal thoughts for a day, what would that day look like?"
"What is your future self doing if your problems are resolved?"
"Are there any specific life experiences you would like to have if things were different?"

If no goals are listed, use more specific examples:

"How important is your family to you?"
"How important is your career to you?"
"How important are your hobbies to you?"
"How important is religion/spirituality to you?"

If they list 1-3 goals, move on to next question

If they cannot list one or more goals

Skip to question ***2 a)***

1 a) Importance of goals:

For each goal, ask the respondent to answer the following question:

How important is it for you to reach (or continue reaching) this goal on a scale from 0 to 10?

1 b) Achievement of goals: *For each goal, ask the respondent to indicate to what extent they have achieved/reached that goal. Please ask respondents asking the following question:*

Have you reached this goal?

Please indicate to what extent you have already reached/achieved this goal, out of the following options:

I have not reached/achieved this goal at all.
I have partially reached/achieved this goal but still need to make more progress.
I have completely reached/achieved this goal and want to continue doing so.
I have already completely achieved/reached this goal and no longer need to pursue it.

1 c) Underlying higher-level motives for each goal: *For each goal, ask the respondent to answer the prompt questions below until they describe why the goal is important to them (i.e. their underlying higher level motive/reason for the goal). It may not be necessary to use all prompt questions, but use whichever number of the prompt questions is required for the respondent to state a much more general higher-level goal that is the reason for their more specific goal. E.g. if they describe wanting to be kind to their friends, their underlying general higher-level goal might be to be a nice person.*

“Why is [goal] important to you?”

“Why does this help?”

“Why do you like [goal]?

“What would be good about reaching [goal]?”

“If you reached that goal, what would that mean to you?”

Move onto next question once they have been prompted to explain why each goal is important to them, even if they are unable to list any reasons for wanting to reach any of the goals

1 d) Ability to access each goal: *For each goal, ask the respondent to answer the questions below. For i), use one or more of the following prompts as examples after reading question i) to the respondent in order to ensure that they understand the question, and use more of these prompts if the respondent has any difficulties in understanding the question:*

"How easy is it to think of the goal?"

"How easy is it to remember the goal?"

"How easy is it to hold the goal in your mind?"

"How easy is it to concentrate on the goal?"

"How easy is it to visualise the goal?"

i) How easy is it to bring this goal into your mind right now? (0 = not at all, 5 = extremely easy)
ii) How often do you think about this? (more than once a day, once a day, once a week, once a month, once every few months, never)
iii) To what extent does it influence your decisions? (0 = not at all, 5 = very much so)

1 e) Means of reaching each goal: *For each goal, ask the respondent to briefly describe how they would reach this goal using the following prompts:*

"Thinking about your current situation right now, how would you reach this goal?"

"What would need to happen for this to be possible?"

"What steps would you need to take for this to happen?"

"If this is already happening, what makes it possible?"

If they cannot describe any means of reaching any of the goals they have listed

If some means of reaching some of the goals are described

Skip to question ***1 g)***

1 f) Identifying external barriers to reaching each goal: *For each goal, ask the respondent the following questions:*

i) What gets in the way of you reaching this goal?
ii) What would happen if you tried to reach this goal?
iii) What choices do you feel you have at the moment in terms of reaching this goal?

1 g) Impact of suicide on goals: *Before asking the questions on the impact of suicide on the respondent's goals, please state the following in order to mentally prepare them for answering suicide-related questions:*

The next three questions are going to mention suicide, so just answer as best you can and let me know if the questions make you feel uncomfortable at all.

For each goal, ask the respondent to answer the questions below.

i) If you died by suicide, could you still reach this goal? (Yes/No)

Before asking question ii), please state the following:

The following two questions both relate to the impact of suicide on how the goal would be reached by you or anyone else. One question is about how suicide would interfere with reaching the goal, and the other question is about how suicide would help with reaching the goal.

ii) If you died by suicide, how much would your death by suicide interfere with [goal] being reached? (0 = not at all, 5 = very much so)
iii) If you died by suicide, how much would your death by suicide help with [goal] being reached? (0 = not at all, 5 = very much so)

1 h) Goal-related mental imagery: *For each goal, ask the respondent to answer the following question:*

Do any mental images come into your mind when you think of this goal? (Yes/No)

A mental image can be either a picture in your mind or something you hear, feel or smell when you think of the goal.

Yes

No

*Skip to question **2 a)***

1 i) Imagery description: Can you describe the images?

Instructions for interviewer: If any prompts are needed, provide the example of someone who wants to eat a cake might have images in their mind of what the icing would look like and how it would taste.

1 j) Imagery details: *For each goal, ask the respondent to answer the following questions:*

i) How vivid are the images? (0 = not vivid at all, 5 = very vivid)
ii) Do you experience the images as though it is through your own eyes (first person perspective), or as though you are watching yourself in the image (third person perspective), or both? (first/ third/ both)
iii) Are the images voluntary (i.e. do you deliberately imagine them), or involuntary (i.e. do they just come into your mind spontaneously), or can they be both? (voluntary/ involuntary/ both)
iv) Do you ever try to keep the images out of your mind? (Yes/No)
v) To what extent do you try to keep the images out of your mind? (0 = not at all, 5 = every time I experience the images)

If no goals were listed in question 1), move to question 2). Otherwise, skip question 2) and move on to the next section of the assessment.

2 a) Other imagery: Are you experiencing any mental images right now, at this moment?

Yes

No → *End this section of the assessment*

2 b) Imagery description: Can you describe the images?

Instructions for interviewer: If any prompts are needed, provide the example of someone who wants to eat a cake might have images in their mind of what the icing would look like and how it would taste.

2 c) Imagery details: *For each image, ask the respondent to answer the following questions:*

i) How vivid is the image? (0 = not vivid at all, 5 = very vivid)
ii) Do you experience the image as though it is through your own eyes (first person perspective), or as though you are watching yourself in the image (third person perspective), or both? (first/ third/ both)
iii) Is the image voluntary (i.e. do you deliberately imagine it), or involuntary (i.e. does it just come into your mind spontaneously), or can it be both? (voluntary/ involuntary/ both)
iv) Do you ever try to keep the image out of your mind? (Yes/No)
v) To what extent do you try to keep the image out of your mind? (0 = not at all, 5 = every time I experience the image)

2 d) Goals which come to mind when focusing on imagery: *For each image, ask the respondent to answer the following question:*

As you focus on the image you have just described, do any goals come to mind that you have, which did not come into your mind during the previous questions about goals?

If yes, repeat questions 1a) – 1g) for each goal listed while responding to this question

If no, move onto Section 2 if applicable

Mood state: *How would you describe your mood at the time you most recently contemplated suicide?*

The interviewer should read out the words from the answer booklet which describe different feelings and emotions, or show them to the respondent. The interviewer should then mark the appropriate answer in the space next to each word, once the respondent has indicated to what extent they felt that way at the time they most recently contemplated suicide.

Section 2 – Awareness of goals at the time they most recently contemplated suicide

Instructions for interviewer: This section follows a similar structure to Section 1, but should focus on the specific occasion when the respondent most recently contemplated suicide.

1) Ability to access each goal listed in Section 1: *For each goal, ask the respondent to answer the questions below.*

i) How often did you think about the goal at that time? (more than once a day, once a day, once a week, once a month, once every few months, never)

For ii), use one or more of the following prompts as examples after reading question ii) to the respondent in order to ensure that they understand the question, and use more of these prompts if the respondent has any difficulties in understanding the question:

"How easy was it to think of the goal?"
"How easy was it to remember the goal?"
"How easy was it to hold the goal in your mind?"
"How easy was it to concentrate on the goal?"
"How easy was it to visualise the goal?"

ii) How easy was it to bring this goal into your mind at that time? (0 = not at all, 5 = very much so)
iii) To what extent did it influence your decisions at that time? (0 = not at all, 5 = very much so)

2) Goals: Can you list one or more goals that are important to you, which came into your mind when you most recently contemplated suicide?

In this assessment, the term "goals" has a broader definition than the one you might normally use. By "goals" we mean future experiences that people generally try to achieve or accomplish (for example, have a successful career, get a promotion at work, be a good parent) as well as experiences that people generally try to avoid (for example, someone could have a goal to feel less lonely or to stop feeling distressed). Goals can be related to something you are already doing, and you want to keep doing (for example, you might have a good relationship with your family members that you want to maintain, or you might want to continue doing well at your job or university work). Goals can be either very specific, such as something that you would like to do or achieve (e.g. go for a holiday in Majorca or pass your driving test) or something specific that you would want to avoid (e.g. being late for an appointment or having an argument with a friend), and they can be either long-term or short-term. Goals can also be more general, such as a goal to help people or be a nice person, or a goal to avoid being a bad person.

If the participant lists more than 3 goals, ask the following question, and then write the 3 goals they answer with in the goals section of the answer booklet:

Which three of these goals are most relevant in your life or do you spend the most time focusing on?

If they list 1-3 goals, move on to next question

If they cannot list one or more goals, use the following prompts

“What is living about for you?”
“Can you give an example of what you do on an everyday basis?”
“Can you picture things you would be doing if things were going right for you?”
“If you could wave a magic wand and not have suicidal thoughts for a day, what would that day look like?”
“What is your future self doing if your problems are resolved?”
“Are there any specific life experiences you would like to have if things were different?"

If no goals are listed, use more specific examples:

“How important is your family to you?”
“How important is your career to you?”
“How important are your hobbies to you?”
“How important is religion/spirituality to you?”

If they list 1-3 goals, move on to next question

If they cannot list one or more goals

Skip to question ***3 a)***

2 a) Importance of goals: *For each goal, ask the respondent to answer the following question:*

How important is it for you to reach this goal now on a scale from 0 to 10?

If the goal is no longer important to them for any reason (e.g. it is a very specific goal which they have now reached), use the probe questions below to find out the more general higher-level goal underlying the more specifc goal. Once they have stated the more general higher-level goal, ask them to rate it from 0 to 10.

“Why was [goal] important to you?”
“Why did it help?”
“Why did you like [goal]?
“What was good about reaching [goal]?”
“What did reaching [goal] mean to you?"

2 b) Achievement of goals: *For each goal, ask the respondent to indicate to what extent they have achieved/reached that goal. Please ask respondents asking the following question:*

*For any goals which are the same as the goals listed in Section 1, please skip to question **2 g)**.*

Have you reached this goal?

Please indicate to what extent you have already reached/achieved this goal, out of the following options:

I have not reached/achieved this goal at all.
I have partially reached/achieved this goal but still need to make more progress.
I have completely reached/achieved this goal and want to continue doing so.
I have already completely achieved/reached this goal and no longer need to pursue it.

2 c) Underlying higher-level motives for each goal: *For each goal, ask the respondent to answer the prompt questions below until they describe why the goal is important to them (i.e. their underlying higher level motive/reason for the goal). It may not be necessary to use all prompt questions, but use whichever number of the prompt questions is required for the respondent to state a much more general higher-level goal that is the reason for their more specific goal. E.g. if they describe wanting to be kind to their friends, their underlying general higher-level goal might be to be a nice person.*

If the goal which they have stated is no longer important to them (e.g. if it was a short-term goal which has already been achieved), please ask the respondent why it was important to them at the time, and use the prompt questions below in the same way to prompt them to describe their underlying motives for the goal.

"Why is [goal] important to you?"

"Why does this help?"

"Why do you like [goal]?

"What would be good about reaching [goal]?"

"If you reached that goal, what would that mean to you?"

Move onto next question once they have been prompted to explain why each goal is important to them, even if they are unable to list any reasons for wanting to reach any of the goals

2 d) Ability to access each goal: *For each goal, ask the respondent to answer the questions below.*

i) How often did you think about the goal at that time? (more than once a day, once a day, once a week, once a month, once every few months, never)

For ii), use one or more of the following prompts as examples after reading question ii) to the respondent in order to ensure that they understand the question, and use more of these prompts if the respondent has difficulties in understanding the question:

"How easy was it to think of the goal?"
"How easy was it to remember the goal?"
"How easy was it to hold the goal in your mind?"
"How easy was it to concentrate on the goal?"
"How easy was it to visualise the goal?"

ii) How easy was it to bring this goal into your mind at that time? (0 = not at all, 5 = very much so)
iii) To what extent did it influence your decisions at that time? (0 = not at all, 5 = very much so)

2 e) Means of reaching each goal: *For each goal, ask the respondent to briefly describe how they would reach this goal using the following prompts.*

"Thinking about your current situation right now, how would you reach this goal?"

"What would need to happen for this to be possible?"

"What steps would you need to take for this to happen?"

"If this is already happening, what makes it possible?"

If they cannot describe any means of reaching any of the goals they have listed → 2 f)

If some means of reaching some of the goals are described → *Skip to question* ***2 g)***

2 f) Identifying external barriers to reaching each goal: *For each goal, ask the respondent the following questions:*

i) What got in the way of you reaching this goal at the time you most recently contemplated suicide?
ii) What would have happened if you had tried to reach this goal around the time that you most recently contemplated suicide?
iii) What choices did you feel you had in terms of reaching this goal, at the time you most recently contemplated suicide?

2 g) Impact of suicide on goals: *Before asking the questions on the impact of suicide on the respondent's goals, please state the following in order to mentally prepare them for answering suicide-related questions:*

The next three questions are going to mention suicide, so just answer as best you can and let me know if the questions make you feel uncomfortable at all.

For each goal, ask the respondent to answer the questions below.

i) At that time, did you feel that this goal could still be reached if you died by suicide? (Yes/No)

Before asking question ii), please state the following:

The following two questions both relate to the impact of suicide on how the goal would be reached by you or anyone else. One question is about how suicide would interfere with reaching the goal, and the other question is about how suicide would help with reaching the goal.

ii) At that time, how much did you feel that dying by suicide would interfere with [goal] being reached? (0 = not at all to 5 = very much so)
iii) At that time, how much did you feel that dying by suicide would help with [goal] being reached? (0 = not at all to 5 = very much so)

2 h) Goal-related mental imagery: *For each goal, ask the respondent to answer the following question:*

If you thought of this goal when you most recently contemplated suicide, did any mental images come into your mind when you thought of it? (Yes/No/Not applicable – did not think of it)

A mental image can be either a picture in your mind or something you hear, feel or smell when you think of the goal.

Yes

No

Skip to question ***3 a)***

2 i) Imagery description: Can you describe the images?

Instructions for interviewer: If any prompts are needed, provide the example of someone who wants to eat a cake might have images in their mind of what the icing would look like and how it would taste.

2 j) Imagery details: *For each goal, ask the respondent to answer the following questions:*

i) How vivid were the images? (0 = not vivid at all, 5 = very vivid)
ii) Did you experience the images as though it was through your own eyes (first person perspective), or as though you were watching yourself in the image (third person perspective), or both? (first/ third/ both)
iii) Were the images voluntary (i.e. did you deliberately imagine them), or involuntary (i.e. did they just come into your mind spontaneously), or could they be both? (voluntary/ involuntary/ both)
iv) Did you ever try to keep the images out of your mind? (Yes/No)
v) To what extent did you try to keep the images out of your mind? (0 = not at all, 5 = every time I experienced the images)

If no goals were listed in question 1), move to question 2). Otherwise, skip question 2) and move on to the next section of the assessment.

3 a) Other imagery: Did any other mental images come into your mind at that time, which are not related to the goals you listed in this section?

Yes

No

End this section of the assessment

3 b) Imagery description: Can you describe the images?

Instructions for interviewer: If any prompts are needed, provide the example of someone who wants to eat a cake might have images in their mind of what the icing would look like and how it would taste.

3 c) Imagery details: *For each image, ask the respondent to answer the following questions:*

i) How vivid was the image? (0 = not vivid at all, 5 = very vivid)
ii) Did you experience the image as though it was through your own eyes (first person perspective), or as though you were watching yourself in the image (third person perspective), or both? (first/ third/ both)
iii) Was the image voluntary (i.e. did you deliberately imagine it), or involuntary (i.e. did it just come into your mind spontaneously), or could it be both? (voluntary/ involuntary/ both)
iv) Did you ever try to keep the image out of your mind? (Yes/No)
v) To what extent did you try to keep the image out of your mind? (0 = not at all, 5 = every time I experienced the image)

3 d) Goals which come to mind when focusing on imagery: *For each image, ask the respondent to answer the following question:*

As you focus on the image you have just described now, do any goals come to mind that you have, which did not come into your mind during the previous questions about goals?

Yes

Repeat questions 2a) – 2g) for each goal listed in the response to this question

No

End this section of the assessment
