# Supplementary material for: Development of a tool for assessing awareness of consequences of suicide
Source: Front Psychol. 2026 Feb 16;17:1736232. doi: 10.3389/fpsyg.2026.1736232 (PMC12950565; doi:10.3389/fpsyg.2026.1736232)
Supplement: Supplementary file 7 [file Data_Sheet_7.pdf]

Supplementary Data Sheet 7: Answer booklet for the final version of the Awareness Assessment Tool

Awareness Assessment Tool answer booklet

The questions in this part of the Awareness Assessment Tool relate to your experiences right now in the present.

Current mood state[1]

What is your current mood at the present moment?

Indicate the extent you feel the following emotions right now, that is, at the present moment	Very slightly or not at all - 1	A little - 2	Moderately - 3	Quite a bit - 4	Extremely - 5
Interested					
Distressed					
Excited					
Upset					
Strong					
Guilty					
Scared					
Hostile					
Enthusiastic					
Proud					
Irritable					

Alert					
Ashamed					
Inspired					
Nervous					
Determined					
Attentive					
Jittery					
Active					
Afraid					

[1]Adapted from Watson, D., Clark, L. A., & Tellegen, A. (1988). Development and validation of brief measures of positive and negative affect: the PANAS scales. *Journal of personality and social psychology*, *54*(6), 1063.

Section 1 – Awareness of goals at the present time

1) Goals

Can you list one or more goals that are important to you?

**Goal**	**Importance (0-10)**
**1)**	

1 b) Have you reached this goal?

Please indicate to what extent you have already reached/achieved this goal:

☐ I have not reached/achieved this goal at all.

☐ I have partially reached/achieved this goal but still need to make more progress.

☐ I have completely reached/achieved this goal and want to continue doing so.

☐ I have already completely achieved/reached this goal and no longer need to pursue it.

1 c) Underlying higher-level motives for each goal:

Why is this important to you?

1 d) Ability to access each goal:

i) How easy is it to bring this goal into your mind right now? (0 = not at all to 5 = extremely easy)

☐

ii) How often do you think about this?

☐ More than once a day

☐ Once a day

☐ Once a week

☐ Once a month

☐ Once every few months

☐ Never

iii) To what extent does it influence your decisions? (0 = not at all to 5 = very much so)

☐

1 e) Means of reaching this goal (subgoals):

Thinking about your current situation right now, how would you reach this goal?

1 f) Identifying external barriers to reaching each goal:

i) What gets in the way of you reaching this goal?

ii) What would happen if you tried to reach this goal?

iii) What choices do you feel you have at the moment in terms of reaching this goal?

1 g) Impact of suicide on goals:

i) If you died by suicide, could you still reach this goal? (Yes/No)

ii) If you died by suicide, how much would your death by suicide interfere with this goal being reached? (0 = not at all to 5 = very much so)

iii) If you died by suicide, how much would your death by suicide help with this goal being reached? (0 = not at all to 5 = very much so)

1 h) Goal-related mental imagery:

Do any mental images come into your mind when you think of this goal? (Yes/No)

A mental image can be either a picture in your mind or something you hear, feel or smell when you think of the goal.

☐

1 i) Imagery description:

Can you describe the images?

1 j) Imagery details:

i) How vivid are the images? (0 = not vivid at all to 5 = very vivid)

☐

ii) Do you experience the images as though it is through your own eyes (first person perspective), or as though you are watching yourself in the image (third person perspective), or both?

☐ The images are always first person perspective.

☐ The images are always third person perspective.

☐ The images can be either first or third person perspective.

iii) Are the images voluntary (i.e. do you deliberately imagine them), or involuntary (i.e. do they just come into your mind spontaneously), or can they be both?

☐ The images are always voluntary.

☐ The images are always involuntary.

☐ The images can be either voluntary or involuntary.

iv) Do you ever try to keep the images out of your mind? (Yes/No)

☐

v) To what extent do you try to keep the images out of your mind? (0 = not at all to 5 = every time I experience the images)

☐

**Goal**	**Importance (0-10)**
**2)**	

1 b) Have you reached this goal?

Please indicate to what extent you have already reached/achieved this goal:

☐ I have not reached/achieved this goal at all.

☐ I have partially reached/achieved this goal but still need to make more progress.

☐ I have completely reached/achieved this goal and want to continue doing so.

☐ I have already completely achieved/reached this goal and no longer need to pursue it.

1 c) Underlying higher-level motives for each goal:

Why is this important to you?

1 d) Ability to access each goal:

i) How easy is it to bring this goal into your mind right now? (0 = not at all to 5 = extremely easy)

☐

ii) How often do you think about this?

- [ ] More than once a day
- [ ] Once a day
- [ ] Once a week
- [ ] Once a month
- [ ] Once every few months
- [ ] Never

iii) To what extent does it influence your decisions? (0 = not at all to 5 = very much so)

[ ]

1 e) Means of reaching this goal (subgoals):

Thinking about your current situation right now, how would you reach this goal?

1 f) Identifying external barriers to reaching each goal:

i) What gets in the way of you reaching this goal?

ii) What would happen if you tried to reach this goal?

iii) What choices do you feel you have at the moment in terms of reaching this goal?

1 g) Impact of suicide on goals:

i) If you died by suicide, could you still reach this goal? (Yes/No)

ii) If you died by suicide, how much would your death by suicide interfere with this goal being reached? (0 = not at all to 5 = very much so)

iii) If you died by suicide, how much would your death by suicide help with this goal being reached? (0 = not at all to 5 = very much so)

1 h) Goal-related mental imagery:

Do any mental images come into your mind when you think of this goal? (Yes/No)

A mental image can be either a picture in your mind or something you hear, feel or smell when you think of the goal.

☐

1 i) Imagery description:

Can you describe the images?

1 j) Imagery details:

i) How vivid are the images? (0 = not vivid at all to 5 = very vivid)

☐

ii) Do you experience the images as though it is through your own eyes (first person perspective), or as though you are watching yourself in the image (third person perspective), or both?

☐ The images are always first person perspective.

☐ The images are always third person perspective.

☐ The images can be either first or third person perspective.

iii) Are the images voluntary (i.e. do you deliberately imagine them), or involuntary (i.e. do they just come into your mind spontaneously), or can they be both?

☐ The images are always voluntary.

☐ The images are always involuntary.

☐ The images can be either voluntary or involuntary.

iv) Do you ever try to keep the images out of your mind? (Yes/No)

☐

v) To what extent do you try to keep the images out of your mind? (0 = not at all to 5 = every time I experience the images)

☐

Goal	Importance (0-10)
3)	

1 b) Have you reached this goal?

Please indicate to what extent you have already reached/achieved this goal:

☐ I have not reached/achieved this goal at all.

☐ I have partially reached/achieved this goal but still need to make more progress.

☐ I have completely reached/achieved this goal and want to continue doing so.

☐ I have already completely achieved/reached this goal and no longer need to pursue it.

1 c) Underlying higher-level motives for each goal:

Why is this important to you?

1 d) Ability to access each goal:

i) How easy is it to bring this goal into your mind right now? (0 = not at all to 5 = extremely easy)

☐

ii) How often do you think about this?

- [ ] More than once a day
- [ ] Once a day
- [ ] Once a week
- [ ] Once a month
- [ ] Once every few months
- [ ] Never

iii) To what extent does it influence your decisions? (0 = not at all to 5 = very much so)

[ ]

1 e) Means of reaching this goal (subgoals):

Thinking about your current situation right now, how would you reach this goal?

1 f) Identifying external barriers to reaching each goal:

i) What gets in the way of you reaching this goal?

ii) What would happen if you tried to reach this goal?

iii) What choices do you feel you have at the moment in terms of reaching this goal?

1 g) Impact of suicide on goals:

i) If you died by suicide, could you still reach this goal? (Yes/No)

ii) If you died by suicide, how much would your death by suicide interfere with this goal being reached? (0 = not at all to 5 = very much so)

iii) If you died by suicide, how much would your death by suicide help with this goal being reached? (0 = not at all to 5 = very much so)

1 h) Goal-related mental imagery:

Do any mental images come into your mind when you think of this goal? (Yes/No)

A mental image can be either a picture in your mind or something you hear, feel or smell when you think of the goal.

☐

1 i) Imagery description:

Can you describe the images?

1 j) Imagery details:

i) How vivid are the images? (0 = not vivid at all to 5 = very vivid)

☐

ii) Do you experience the images as though it is through your own eyes (first person perspective), or as though you are watching yourself in the image (third person perspective), or both?

☐ The images are always first person perspective.

☐ The images are always third person perspective.

☐ The images can be either first or third person perspective.

iii) Are the images voluntary (i.e. do you deliberately imagine them), or involuntary (i.e. do they just come into your mind spontaneously), or can they be both?

☐ The images are always voluntary.

☐ The images are always involuntary.

☐ The images can be either voluntary or involuntary.

iv) Do you ever try to keep the images out of your mind? (Yes/No)

☐

v) To what extent do you try to keep the images out of your mind? (0 = not at all to 5 = every time I experience the images)

☐

2 a) Other mental imagery:

Are you experiencing any mental images right now, at this moment? (Yes/No)

2 b) Imagery description:

Can you describe the images?

2 c) Imagery details:

First image:

i) How vivid is the image? (0 = not vivid at all to 5 = very vivid)

ii) Do you experience the image as though it is through your own eyes (first person perspective), or as though you are watching yourself in the image (third person perspective), or both?

☐ The image is always first person perspective.

☐ The image is always third person perspective.

☐ The image can be either first or third person perspective.

iii) Is the image voluntary (i.e. do you deliberately imagine it), or involuntary (i.e. does it just come into your mind spontaneously), or can it be both?

☐ The image is always voluntary.

☐ The image is always involuntary.

☐ The image can be either voluntary or involuntary.

iv) Do you ever try to keep the image out of your mind? (Yes/No)

☐

v) To what extent do you try to keep the image out of your mind? (0 = not at all to 5 = every time I experience the image)

☐

Second image:

i) How vivid is the image? (0 = not vivid at all to 5 = very vivid)

☐

ii) Do you experience the image as though it is through your own eyes (first person perspective), or as though you are watching yourself in the image (third person perspective), or both?

☐ The image is always first person perspective.

☐ The image is always third person perspective.

☐ The image can be either first or third person perspective.

iii) Is the image voluntary (i.e. do you deliberately imagine it), or involuntary (i.e. does it just come into your mind spontaneously), or can it be both?

☐ The image is always voluntary.

☐ The image is always involuntary.

☐ The image can be either voluntary or involuntary.

iv) Do you ever try to keep the image out of your mind? (Yes/No)

☐

v) To what extent do you try to keep the image out of your mind? (0 = not at all to 5 = every time I experience the image)

☐

Third image:

i) How vivid is the image? (0 = not vivid at all to 5 = very vivid)

☐

ii) Do you experience the image as though it is through your own eyes (first person perspective), or as though you are watching yourself in the image (third person perspective), or both?

☐ The image is always first person perspective.

☐ The image is always third person perspective.

☐ The image can be either first or third person perspective.

iii) Is the image voluntary (i.e. do you deliberately imagine it), or involuntary (i.e. does it just come into your mind spontaneously), or can it be both?

☐ The image is always voluntary.

☐ The image is always involuntary.

☐ The image can be either voluntary or involuntary.

iv) Do you ever try to keep the image out of your mind? (Yes/No)

☐

v) To what extent do you try to keep the image out of your mind? (0 = not at all to 5 = every time I experience the image)

☐

Fourth image:

i) How vivid is the image? (0 = not vivid at all to 5 = very vivid)

☐

ii) Do you experience the image as though it is through your own eyes (first person perspective), or as though you are watching yourself in the image (third person perspective), or both?

☐ The image is always first person perspective.

☐ The image is always third person perspective.

☐ The image can be either first or third person perspective.

iii) Is the image voluntary (i.e. do you deliberately imagine it), or involuntary (i.e. does it just come into your mind spontaneously), or can it be both?

☐ The image is always voluntary.

☐ The image is always involuntary.

☐ The image can be either voluntary or involuntary.

iv) Do you ever try to keep the image out of your mind? (Yes/No)

☐

v) To what extent do you try to keep the image out of your mind? (0 = not at all to 5 = every time I experience the image)

☐

Fifth image:

i) How vivid is the image? (0 = not vivid at all to 5 = very vivid)

☐

ii) Do you experience the image as though it is through your own eyes (first person perspective), or as though you are watching yourself in the image (third person perspective), or both?

☐ The image is always first person perspective.

☐ The image is always third person perspective.

☐ The image can be either first or third person perspective.

iii) Is the image voluntary (i.e. do you deliberately imagine it), or involuntary (i.e. does it just come into your mind spontaneously), or can it be both?

☐ The image is always voluntary.

☐ The image is always involuntary.

☐ The image can be either voluntary or involuntary.

iv) Do you ever try to keep the image out of your mind? (Yes/No)

☐

v) To what extent do you try to keep the image out of your mind? (0 = not at all to 5 = every time I experience the image)

☐

The questions from this point onwards relate to your experiences at the time that you last thought about suicide.

Mood state at time of recent suicide contemplation[2]

How would you describe your mood at the time you most recently contemplated suicide?

Indicate the extent you feel the following emotions right now, that is, at the present moment	Very slightly or not at all - 1	A little - 2	Moderately - 3	Quite a bit - 4	Extremely - 5
Interested					
Distressed					
Excited					
Upset					
Strong					
Guilty					
Scared					
Hostile					
Enthusiastic					
Proud					
Irritable					
Alert					
Ashamed					
Inspired					

Nervous					
Determined					
Attentive					
Jittery					
Active					
Afraid					

[2]Adapted from Watson, D., Clark, L. A., & Tellegen, A. (1988). Development and validation of brief measures of positive and negative affect: the PANAS scales. *Journal of personality and social psychology*, *54*(6), 1063.

Section 2 – Awareness of goals at the time they most recently contemplated suicide

1) Ability to access each goal listed in Section 1 during suicide contemplation

Goal 1)

i) How often did you think about the goal at that time? (i.e. when you most recently contemplated suicide)

- ☐ More than once a day
- ☐ Once a day
- ☐ Once a week
- ☐ Once a month
- ☐ Once every few months
- ☐ Never

ii) How easy was it to bring this goal into your mind at that time? (0 = not at all to 5 = very much so)

☐

iii) To what extent did it influence your decisions at that time? (0 = not at all to 5 = very much so)

☐

Goal 2)

i) How often did you think about the goal at that time? (i.e. when you most recently contemplated suicide)

- ☐ More than once a day
- ☐ Once a day
- ☐ Once a week
- ☐ Once a month
- ☐ Once every few months
- ☐ Never

ii) How easy was it to bring this goal into your mind at that time? (0 = not at all to 5 = very much so)

☐

iii) To what extent did it influence your decisions at that time? (0 = not at all to 5 = very much so)

☐

Goal 3)

i) How often did you think about the goal at that time? (i.e. when you most recently contemplated suicide)

- ☐ More than once a day
- ☐ Once a day
- ☐ Once a week

☐ Once a month

☐ Once every few months

☐ Never

ii) How easy was it to bring this goal into your mind at that time? (0 = not at all to 5 = very much so)

☐

iii) To what extent did it influence your decisions at that time? (0 = not at all to 5 = very much so)

☐

2) Goals

Can you list one or more goals that are important to you, which came into your mind when you most recently contemplated suicide?

**Goal**	**Importance (0-10)**
**1)**	

2 b) Have you reached this goal?

Please indicate to what extent you have already reached/achieved this goal:

☐ I have not reached/achieved this goal at all.

☐ I have partially reached/achieved this goal but still need to make more progress.

☐ I have completely reached/achieved this goal and want to continue doing so.

☐ I have already completely achieved/reached this goal and no longer need to pursue it.

2 c) Underlying higher-level motives for each goal:

Why is this important to you now? Or if it is no longer important, why was it important to you then?

2 d) Ability to access each goal:

i) How often did you think about the goal at that time? (i.e. when you most recently contemplated suicide)

- ☐ More than once a day
- ☐ Once a day
- ☐ Once a week
- ☐ Once a month
- ☐ Once every few months
- ☐ Never

ii) How easy was it to bring this goal into your mind at that time? (0 = not at all to 5 = very much so)

☐

iii) To what extent did it influence your decisions at that time? (0 = not at all to 5 = very much so)

☐

2 e) Means of reaching this goal (subgoals):

Thinking about your current situation right now, how would you reach this goal?

2 f) Identifying external barriers to reaching each goal:

i) What got in the way of you reaching this goal at the time you most recently contemplated suicide?

ii) What would have happened if you had tried to reach this goal around the time that you most recently contemplated suicide?

iii) What choices did you feel you had in terms of reaching this goal, at the time you most recently contemplated suicide?

2 g) Impact of suicide on goals:

i) At that time, did you feel that this goal could still be reached if you died by suicide? (Yes/No)

ii) At that time, how much did you feel that dying by suicide would interfere with this goal being reached? (0 = not at all to 5 = very much so)

iii) At that time, how much did you feel that dying by suicide would help with this goal being reached? (0 = not at all to 5 = very much so)

2 h) Goal-related mental imagery:

If you thought of this goal when you most recently contemplated suicide, did any mental images come into your mind when you thought of it? (Yes/No/Not applicable – did not think of it)

A mental image can be either a picture in your mind or something you heard, felt or smelled when you thought of the goal.

☐

2 i) Imagery description:

Can you describe the images?

2 j) Imagery details:

i) How vivid were the images? (0 = not vivid at all to 5 = very vivid)

☐

ii) Did you experience the images as though it was through your own eyes (first person perspective), or as though you were watching yourself in the image (third person perspective), or both?

☐ The images were always first person perspective.

☐ The images were always third person perspective.

☐ The images could be either first or third person perspective.

iii) Were the images voluntary (i.e. did you deliberately imagine them), or involuntary (i.e. did they just come into your mind spontaneously), or could they be both?

☐ The images were always voluntary.

☐ The images were always involuntary.

☐ The images could be either voluntary or involuntary.

iv) Did you ever try to keep the images out of your mind? (Yes/No)

☐

v) To what extent did you try to keep the images out of your mind? (0 = not at all to 5 = every time I experienced the images)

☐

Goal	Importance (0-10)
2)	

2 b) Have you reached this goal?

Please indicate to what extent you have already reached/achieved this goal:

☐ I have not reached/achieved this goal at all.

☐ I have partially reached/achieved this goal but still need to make more progress.

☐ I have completely reached/achieved this goal and want to continue doing so.

☐ I have already completely achieved/reached this goal and no longer need to pursue it.

2 c) Underlying higher-level motives for each goal:

Why is this important to you now? Or if it is no longer important, why was it important to you then?

2 d) Ability to access each goal:

i) How often did you think about the goal at that time? (i.e. when you most recently contemplated suicide)

☐ More than once a day

☐ Once a day

☐ Once a week

☐ Once a month

☐ Once every few months

☐ Never

ii) How easy was it to bring this goal into your mind at that time? (0 = not at all to 5 = very much so)

☐

iii) To what extent did it influence your decisions at that time? (0 = not at all to 5 = very much so)

☐

2 e) Means of reaching this goal (subgoals):

Thinking about your current situation right now, how would you reach this goal?

2 f) Identifying external barriers to reaching each goal:

i) What got in the way of you reaching this goal at the time you most recently contemplated suicide?

ii) What would have happened if you had tried to reach this goal around the time that you most recently contemplated suicide?

iii) What choices did you feel you had in terms of reaching this goal, at the time you most recently contemplated suicide?

2 g) Impact of suicide on goals:

i) At that time, did you feel that this goal could still be reached if you died by suicide? (Yes/No)

ii) At that time, how much did you feel that dying by suicide would interfere with this goal being reached? (0 = not at all to 5 = very much so)

iii) At that time, how much did you feel that dying by suicide would help with this goal being reached? (0 = not at all to 5 = very much so)

☐

2 h) Goal-related mental imagery:

If you thought of this goal when you most recently contemplated suicide, did any mental images come into your mind when you thought of it? (Yes/No/Not applicable – did not think of it)

A mental image can be either a picture in your mind or something you heard, felt or smelled when you thought of the goal.

☐

2 i) Imagery description:

Can you describe the images?

2 j) Imagery details:

i) How vivid were the images? (0 = not vivid at all to 5 = very vivid)

☐

ii) Did you experience the images as though it was through your own eyes (first person perspective), or as though you were watching yourself in the image (third person perspective), or both?

☐ The images were always first person perspective.

☐ The images were always third person perspective.

☐ The images could be either first or third person perspective.

iii) Were the images voluntary (i.e. did you deliberately imagine them), or involuntary (i.e. did they just come into your mind spontaneously), or could they be both?

☐ The images were always voluntary.

☐ The images were always involuntary.

☐ The images could be either voluntary or involuntary.

iv) Did you ever try to keep the images out of your mind? (Yes/No)

☐

v) To what extent did you try to keep the images out of your mind? (0 = not at all to 5 = every time I experienced the images)

☐

Goal	Importance (0-10)
3)	

2 b) Have you reached this goal?

Please indicate to what extent you have already reached/achieved this goal:

☐ I have not reached/achieved this goal at all.

☐ I have partially reached/achieved this goal but still need to make more progress.

☐ I have completely reached/achieved this goal and want to continue doing so.

☐ I have already completely achieved/reached this goal and no longer need to pursue it.

2 c) Underlying higher-level motives for each goal:

Why is this important to you now? Or if it is no longer important, why was it important to you then?

2 d) Ability to access each goal:

i) How often did you think about the goal at that time? (i.e. when you most recently contemplated suicide)

☐ More than once a day

☐ Once a day

☐ Once a week

☐ Once a month

☐ Once every few months

☐ Never

ii) How easy was it to bring this goal into your mind at that time? (0 = not at all to 5 = very much so)

☐

iii) To what extent did it influence your decisions at that time? (0 = not at all to 5 = very much so)

☐

2 e) Means of reaching this goal (subgoals):

Thinking about your current situation right now, how would you reach this goal?

2 f) Identifying external barriers to reaching each goal:

i) What got in the way of you reaching this goal at the time you most recently contemplated suicide?

ii) What would have happened if you had tried to reach this goal around the time that you most recently contemplated suicide?

iii) What choices did you feel you had in terms of reaching this goal, at the time you most recently contemplated suicide?

2 g) Impact of suicide on goals:

i) At that time, did you feel that this goal could still be reached if you died by suicide? (Yes/No)

ii) At that time, how much did you feel that dying by suicide would interfere with this goal being reached? (0 = not at all to 5 = very much so)

iii) At that time, how much did you feel that dying by suicide would help with this goal being reached? (0 = not at all to 5 = very much so)

☐

2 h) Goal-related mental imagery:

If you thought of this goal when you most recently contemplated suicide, did any mental images come into your mind when you thought of it? (Yes/No/Not applicable – did not think of it)

A mental image can be either a picture in your mind or something you heard, felt or smelled when you thought of the goal.

☐

2 i) Imagery description:

Can you describe the images?

2 j) Imagery details:

i) How vivid were the images? (0 = not vivid at all to 5 = very vivid)

☐

ii) Did you experience the images as though it was through your own eyes (first person perspective), or as though you were watching yourself in the image (third person perspective), or both?

☐ The images were always first person perspective.

☐ The images were always third person perspective.

☐ The images could be either first or third person perspective.

iii) Were the images voluntary (i.e. did you deliberately imagine them), or involuntary (i.e. did they just come into your mind spontaneously), or could they be both?

☐ The images were always voluntary.

☐ The images were always involuntary.

☐ The images could be either voluntary or involuntary.

iv) Did you ever try to keep the images out of your mind? (Yes/No)

☐

v) To what extent did you try to keep the images out of your mind? (0 = not at all to 5 = every time I experienced the images)

☐

3 a) Other mental imagery:

Did any other mental images come into your mind when you most recently contemplated suicide, which are **not** related to the goals you listed in this section? (Yes/No)

☐

3 b) Imagery description:

Can you describe the images?

3 c) Imagery details:

First image:

i) How vivid was the image? (0 = not vivid at all to 5 = very vivid)

ii) Did you experience the image as though it was through your own eyes (first person perspective), or as though you were watching yourself in the image (third person perspective), or both?

☐ The image was always first person perspective.

☐ The image was always third person perspective.

☐ The image could be either first or third person perspective.

iii) Was the image voluntary (i.e. did you deliberately imagine it), or involuntary (i.e. did it just come into your mind spontaneously), or could it be both?

☐ The image was always voluntary.

☐ The image was always involuntary.

☐ The image could be either voluntary or involuntary.

iv) Did you ever try to keep the image out of your mind? (Yes/No)

☐

v) To what extent did you try to keep the image out of your mind? (0 = not at all to 5 = every time I experienced the image)

☐

Second image:

i) How vivid was the image? (0 = not vivid at all to 5 = very vivid)

☐

ii) Did you experience the image as though it was through your own eyes (first person perspective), or as though you were watching yourself in the image (third person perspective), or both?

☐ The image was always first person perspective.

☐ The image was always third person perspective.

☐ The image could be either first or third person perspective.

iii) Was the image voluntary (i.e. did you deliberately imagine it), or involuntary (i.e. did it just come into your mind spontaneously), or could it be both?

☐ The image was always voluntary.

☐ The image was always involuntary.

☐ The image could be either voluntary or involuntary.

iv) Did you ever try to keep the image out of your mind? (Yes/No)

☐

v) To what extent did you try to keep the image out of your mind? (0 = not at all to 5 = every time I experienced the image)

☐

Third image:

i) How vivid was the image? (0 = not vivid at all to 5 = very vivid)

☐

ii) Did you experience the image as though it was through your own eyes (first person perspective), or as though you were watching yourself in the image (third person perspective), or both?

☐ The image was always first person perspective.

☐ The image was always third person perspective.

☐ The image could be either first or third person perspective.

iii) Was the image voluntary (i.e. did you deliberately imagine it), or involuntary (i.e. did it just come into your mind spontaneously), or could it be both?

☐ The image was always voluntary.

☐ The image was always involuntary.

☐ The image could be either voluntary or involuntary.

iv) Did you ever try to keep the image out of your mind? (Yes/No)

☐

v) To what extent did you try to keep the image out of your mind? (0 = not at all to 5 = every time I experienced the image)

☐

Fourth image:

i) How vivid was the image? (0 = not vivid at all to 5 = very vivid)

☐

ii) Did you experience the image as though it was through your own eyes (first person perspective), or as though you were watching yourself in the image (third person perspective), or both?

☐ The image was always first person perspective.

☐ The image was always third person perspective.

☐ The image could be either first or third person perspective.

iii) Was the image voluntary (i.e. did you deliberately imagine it), or involuntary (i.e. did it just come into your mind spontaneously), or could it be both?

☐ The image was always voluntary.

☐ The image was always involuntary.

☐ The image could be either voluntary or involuntary.

iv) Did you ever try to keep the image out of your mind? (Yes/No)

☐

v) To what extent did you try to keep the image out of your mind? (0 = not at all to 5 = every time I experienced the image)

☐

Fifth image:

i) How vivid was the image? (0 = not vivid at all to 5 = very vivid)

☐

ii) Did you experience the image as though it was through your own eyes (first person perspective), or as though you were watching yourself in the image (third person perspective), or both?

☐ The image was always first person perspective.

☐ The image was always third person perspective.

☐ The image could be either first or third person perspective.

iii) Was the image voluntary (i.e. did you deliberately imagine it), or involuntary (i.e. did it just come into your mind spontaneously), or could it be both?

☐ The image was always voluntary.

☐ The image was always involuntary.

☐ The image could be either voluntary or involuntary.

iv) Did you ever try to keep the image out of your mind? (Yes/No)

☐

v) To what extent did you try to keep the image out of your mind? (0 = not at all to 5 = every time I experienced the image)

☐
