# Supplementary material for: Development of a tool for assessing awareness of consequences of suicide
Source: Front Psychol. 2026 Feb 16;17:1736232. doi: 10.3389/fpsyg.2026.1736232 (PMC12950565; doi:10.3389/fpsyg.2026.1736232)
Supplement: Supplementary file 8 [file Table_1.docx]

Supplementary Table 1: Full list of Awareness Assessment Tool (AAT) refinements during its development

| **Awareness Assessment Tool (AAT) refinement** | **Rationale** |
| --- | --- |
| **First set of AAT refinements** | |
| Text was added to the answer booklet at the beginning of each section (Present Time Experiences Section and Contemplating Suicide Section), to indicate whether that section refers to past or present experiences. | The added text was intended as a prompt for the interviewer to clarify whether the section refers to present experiences or the respondent’s most recent suicidal episode. This ensures respondents know which time frame to consider when answering. |
| A citation for the Positive and Negative Affect Schedule (PANAS) (Watson et al., 1988) was added to both AAT sections for the adapted PANAS. | This addition ensured that credit was clearly given to the authors of the original PANAS. |
| Each item from the AAT flowchart was added to the AAT answer booklet. | This addition was intended to improve the AAT’s ease of completion, since it enables the interviewer to show the items in the answer booklet to the respondent if necessary. |
| Interviewer instructions for each AAT section were updated, instructing the interviewer to state that respondents may list both specific and broader goals, with examples provided. | This was intended to provide additional clarification, to ensure that in cases where more specific goals are applicable, respondents feel free to list these goals. |
| In the AAT answer booklet, the total number of goals which respondents can list and complete subsequent items for in each AAT section was reduced from eight to three. | This aimed to improve the feasibility of AAT completion during research interviews, as the AAT takes around 50–60 minutes with three goals for each section and would take longer with eight. |
| The AAT interviewer instructions for both sections were updated to include a prompt to keep probing until the respondent identifies more abstract, higher-level motives for each goal, when responding to the item on underlying higher-level motives for goals. | This change ensured that responses to that item provided an accurate reflection of respondents’ awareness, by ensuring that they listed the highest-level goal which they were able to generate. |
| The phrasing of the first item on ability to access each goal in the Present Time Experiences Section was changed from “How able are you to bring this goal to mind?” to “How easy is it to bring this goal into your mind right now?”. | This phrasing was changed to improve its clarity, following feedback in the current study that the previous phrasing was sometimes unclear. |
| The phrasing of the first item on ability to access each goal in the Contemplating Suicide Section was changed from “How easy did you find it to bring this goal to mind at the time you most recently contemplated suicide?” to “How easy was it to bring this goal into your mind at that time?” | The phrasing and length of this item was changed to improve its clarity, following feedback in the current study that the previous phrasing was sometimes unclear and that it was too long. |
| The following prompts were added to the interviewer instructions for the first item on ability to access each goal for both AAT sections:   - “How easy is it to think of the goal?” - “How easy is it to remember the goal?” - “How easy is it to hold the goal in your mind?” - “How easy is it to concentrate on the goal?” - “How easy is it to visualise the goal?” | These prompts aimed to provide clarification to ensure that respondents fully understand the question. |
| In the Contemplating Suicide Section, the order of the first two items on ability to access goals was changed to the following:   - How often did you think about the goal at that time? - How easy was it to bring this goal into your mind at that time? | This reordering aimed to enhance the ease of responding to the item about how easily the goal came to mind. Prompting prior reflection on how often the goal was considered during suicidal contemplation was expected to facilitate responses to this subsequent question. |
| In the Present Time Experiences Section, the phrasing of the three items asking, “If you died by suicide, how much would it interfere with the achievement of this goal?”, “If you died by suicide, could you still achieve this goal?”, and “If you died by suicide, how much would it help with achieving this goal?” was changed to the following:   - If you died by suicide, could you still reach this goal? - If you died by suicide, how much would it interfere with this goal being reached? - If you died by suicide, how much would it help with this goal being reached? | Using the word 'reach' rather than 'achieve' aimed to encourage respondents to consider goals more broadly – as everyday aims and values consistent with Perceptual Control Theory – rather than solely as long-term accomplishments (e.g., graduation). This was expected to enhance both ease of completion and content validity of these items. |
| In the Contemplating Suicide Section, the phrasing of the items asking, “If you died by suicide, how much would it interfere with the achievement of this goal?”, “If you died by suicide, could you still achieve this”, and “If you died by suicide, how much would it help with achieving this goal?” was changed to the following:   - At that time, did you feel that this goal could still be reached if you died by suicide? - At that time, how much did you feel that dying by suicide would interfere with this goal being reached? - At that time, how much did you feel that dying by suicide would help with reaching this goal? | Using the word 'reach' rather than 'achieve' aimed to encourage respondents to consider goals more broadly – as everyday aims and values consistent with Perceptual Control Theory – rather than solely as long-term accomplishments (e.g., graduation). This was expected to enhance both ease of completion and content validity of these items.  In addition, the focus of these items was shifted from current awareness of how suicide would impact these goals to respondents’ awareness while contemplating suicide. This change was anticipated to enable comparisons between present awareness and awareness while contemplating suicide for these items. |
| The order of the first two questions on impact of suicide were swapped in the Present Time Experiences Section, so that they are in the following order:   - If you died by suicide, could you still reach this goal? (Yes/No) - If you died by suicide, how much would it interfere with this goal being reached? (0 = not at all to 5 = very much so) | This aimed to improve the ease of responding to the item on how much suicide would interfere with goal achievement. Prompting respondents to consider whether the goal could be achieved at all following their death from suicide was expected to encourage reflection on how much suicide would interfere with the goal. |
| The order of the first two questions on impact of suicide were swapped in the Contemplating Suicide Section, so that they are in the following order:   - At that time, did you feel that this goal could still be reached if you died by suicide? (Yes/No) - At that time, how much did you feel that dying by suicide would interfere with this goal being reached? (0 = not at all to 5 = very much so) | This aimed to improve the ease of responding to the item on how much suicide would interfere with goal achievement. Prompting respondents to consider whether the goal could be achieved at all following their death from suicide was expected to encourage reflection on how much suicide would interfere with the goal. |
| In both AAT sections, for the question on whether respondents experienced any mental images when thinking of a particular goal, the following interviewer prompt was added:   - “A mental image can be either a picture in your mind or something you hear, feel or smell when you think of the goal.” | This prompt clarified that images need not be visual, to ensure respondents reported non-visual imagery as well. |
| The first item about goal-related imagery in the Contemplating Suicide Section was changed from “Did any mental images come into your mind when you think of this goal when you most recently contemplated suicide? (Yes/No)” to the following:   - “If you thought of this goal when you most recently contemplated suicide, did any mental images come into your mind when you thought of it? (Yes/No/Not applicable – did not think of it)” | This was changed to account for cases when respondents either did not think of the goal when they most recently contemplated suicide, or did not experience any goal-related mental images. |
| The item on goal-related imagery perspective in the Present Time Experiences section was split into the following two items:   - Do you experience the images as though it is through your own eyes (first person perspective)? (Yes/No) - Do you experience the images as though you are watching yourself in the image (third person perspective)? (Yes/No) | As some participants reported imagery from both first- and third-person perspectives, this change allowed respondents to indicate both, rather than choose one or the other. |
| The item on goal-related imagery perspective in the Contemplating Suicide Section was split into the following two items:   - Did you experience the images as though it was through your own eyes (first person perspective)? (Yes/No) - Did you experience the images as though you were watching yourself in the image (third person perspective)? (Yes/No) | As some participants reported imagery from both first- and third-person perspectives, this change allowed respondents to indicate both, rather than choose one or the other. |
| The item on goal-related imagery voluntariness in the Present Time Experiences Section was split into the following two items:   - Are the images ever voluntary (i.e. do you deliberately imagine them) (Yes/No)? - Are the images ever involuntary (i.e. do they just come into your mind spontaneously) (Yes/No)? | As some participants reported some imagery being both voluntary and involuntary, this change allowed respondents to indicate both, rather than choose one or the other. |
| The item on goal-related imagery voluntariness in the Contemplating Suicide Section was split into the following two items:   - Were the images ever voluntary (i.e. did you deliberately imagine them)? (Yes/No) - Were the images ever involuntary (i.e. did they just come into your mind spontaneously)? (Yes/No) | As some participants reported some imagery being both voluntary and involuntary, this change allowed respondents to indicate both, rather than choose one or the other. |
| In the Present Time Experiences Section, the following question was added before the item asking respondents to what extent they try to keep goal-related imagery out of their minds:   - Do you ever try to keep the images out of your mind? (Yes/No) | Some participants were confused by the question about to what extent they keep imagery out of their minds, as it did not apply to them. This item was added so interviewers could skip the next question about the extent which mental imagery is kept out of one’s mind when irrelevant to respondents. |
| In the Contemplating Suicide Section, the following question was added before the item asking respondents to what extent they try to keep goal-related imagery out of their minds:   - Did you ever try to keep the images out of your mind? (Yes/No) | Some participants were confused by the question about to what extent they keep imagery out of their minds, as it did not apply to them. This item was added so interviewers could skip the next question about the extent which mental imagery is kept out of one’s mind when irrelevant to respondents. |
| The questions on visual perspective of other mental imagery in the Present Time Experiences Section were split into the following two questions:   - Do you experience the images as though it is through your own eyes (first person perspective)? (Yes/No) - Do you experience the images as though you are watching yourself in the image (third person perspective)? (Yes/No) | As some participants reported imagery from both first- and third-person perspectives, this change allowed respondents to indicate both, rather than choose one or the other. |
| The questions on visual perspective of other mental imagery in the Contemplating Suicide Section were split into the following two questions:   - Did you experience the images as though it was through your own eyes (first person perspective)? (Yes/No) - Did you experience the images as though you were watching yourself in the image (third person perspective)? (Yes/No) | As some participants reported imagery from both first- and third-person perspectives, this change allowed respondents to indicate both, rather than choose one or the other. |
| The questions on voluntariness of other mental imagery in the Present Time Experiences Section were split into the following two questions:   - Are the images ever voluntary (i.e. do you deliberately imagine them) (Yes/No)? - Are the images ever involuntary (i.e. do they just come into your mind spontaneously) (Yes/No)? | As some participants reported some imagery being both voluntary and involuntary, this change allowed respondents to indicate both, rather than choose one or the other. |
| The questions on voluntariness of other mental imagery in the Contemplating Suicide Section were split into the following two questions:   - Were the images ever voluntary (i.e. did you deliberately imagine them)? (Yes/No) - Were the images ever involuntary (i.e. did they just come into your mind spontaneously)? (Yes/No) | As some participants reported some imagery being both voluntary and involuntary, this change allowed respondents to indicate both, rather than choose one or the other. |
| In the Present Time Experiences Section, the following question was added before the question asking respondents to what extent they try to keep other mental imagery out of their minds:   - Do you ever try to keep the images out of your mind? (Yes/No) | Some participants were confused by the question about to what extent they keep imagery out of their minds, as it did not apply to them. This item was added so interviewers could skip the next question about the extent which mental imagery is kept out of one’s mind when irrelevant to respondents. |
| In the Contemplating Suicide Section, the following question was added before the question asking respondents to what extent they try to keep other mental imagery out of their minds:   - Did you ever try to keep the images out of your mind? (Yes/No) | Some participants were confused by the question about to what extent they keep imagery out of their minds, as it did not apply to them. This item was added so interviewers could skip the next question about the extent which mental imagery is kept out of one’s mind when irrelevant to respondents. |
| In the Contemplating Suicide section on ability to access each goal listed in Section 1 (Present Time Experiences Section) during suicide contemplation, the phrasing of the item, “How easy did you find it to bring this goal to mind at the time you most recently contemplated suicide?” was changed to the following:   - How easy was it to bring this goal into your mind at that time? | Feedback from participants indicated that this item’s length made it difficult to complete, so shortening it was expected to ease completion. |
| In the Contemplating Suicide section on ability to access each goal listed in Section 1 (Present Time Experiences Section) during suicide contemplation, the order of the first two items was changed to the following:   - How often did you think about the goal at that time? (i.e. when you most recently contemplated suicide) - How easy was it to bring this goal into your mind at that time? | This reordering aimed to enhance the ease of responding to the item about how easily the goal came to mind. Prompting prior reflection on how often the goal was considered during suicidal contemplation was expected to facilitate responses to this subsequent question. |
| **Second set of AAT refinements** | |
| Text was added to the flowchart which instructs the interviewer to warn participants that suicide related questions follow next, before the ‘impact of suicide on goals’ items in both AAT sections and before the beginning of the Contemplating Suicide Section. | This text was added because in the current study, a participant gave feedback that people might find it helpful if they are told in advance that they are about to be asked suicide related questions. Therefore, this warning was anticipated to improve the AAT’s acceptability. |
| An item has been added to both AAT sections asking to what extent the respondent’s listed goal has been achieved. | In the current study, some participants had already achieved some of their listed goals. The addition of this new item provided a way of recording this information. This was considered important for distinguishing between goals which are rated as unimportant to respondents because they have already been achieved, and goals which are perceived as unimportant for other reasons. |
| In the Contemplating Suicide Section, the following additional text has been added to the item on underlying higher-level motives for each goal, which asks about the reasons why a goal is important to a respondent:   - Or if it is no longer important, why was it important to you then? | In the current study, some participants had already achieved some of their listed goals. This additional question aimed to gain information about respondents’ awareness of underlying motives for goals, even if the goals are no longer important to them. |
| The ‘impact of suicide on goals’ questions in the Present Time Experiences Section have been edited to have the following phrasing:   - If you died by suicide, how much would your death by suicide interfere with this goal being reached? (0 = not at all to 5 = very much so) - If you died by suicide, how much would your death by suicide help with this goal being reached? (0 = not at all to 5 = very much so) | This aimed to clarify that the AAT was specifically asking about the impact of respondents’ death from suicide, as opposed to any other aspects of suicide. |
| The ‘impact of suicide on goals’ questions in the Contemplating Suicide Section have been edited to have the following phrasing:   - At that time, did you feel that this goal could still be reached if you died by suicide? (Yes/No) - At that time, how much did you feel that dying by suicide would interfere with this goal being reached? (0 = not at all to 5 = very much so) - At that time, how much did you feel that dying by suicide would help with this goal being reached? (0 = not at all to 5 = very much so) | This aimed to clarify that the AAT was specifically asking about the impact of respondents’ death from suicide, as opposed to any other aspects of suicide. |
| The items on whether images are experienced from the first or third person perspective in the Present Time Experiences Section were combined into multiple choice options within the same item. The item now asks:   - Do you experience the images as though it is through your own eyes (first person perspective), or as though you are watching yourself in the image (third person perspective), or both? - The images are always first person perspective. - The images are always third person perspective. - The images can be either first or third person perspective. | These items were revised following reflection by the researcher, who determined that their original phrasing was too closed and potentially leading when presented separately. Combining them into a single multiple-choice item allowed respondents to indicate experiencing both visual perspectives and minimised bias in questioning. |
| The items on whether images are experienced from the first or third person perspective in the Contemplating Suicide Section were combined into multiple choice options within the same item. The item now asks:   - Did you experience the images as though it was through your own eyes (first person perspective), or as though you were watching yourself in the image (third person perspective), or both? - The images were always first person perspective. - The images were always third person perspective. - The images could be either first or third person perspective. | These items were revised following reflection by the researcher, who determined that their original phrasing was too closed and potentially leading when presented separately. Combining them into a single multiple-choice item allowed respondents to indicate experiencing both visual perspectives and minimised bias in questioning. |
| The items on voluntariness of imagery in the Present Time Experiences Section were combined into multiple choice options within the same item. The item now asks:   - Are the images voluntary (i.e. do you deliberately imagine them), or involuntary (i.e. do they just come into your mind spontaneously), or can they be both? - The images are always voluntary. - The images are always involuntary. - The images can be either voluntary or involuntary. | These items were revised following reflection by the researcher, who determined that their original phrasing was too closed and potentially leading when presented separately. Combining them into a single multiple-choice item allowed respondents to indicate experiencing both voluntary and involuntary images and minimised bias in questioning. |
| The items on voluntariness of imagery in the Contemplating Suicide Section were combined into multiple choice options within the same item. The item now asks:   - Were the images voluntary (i.e. did you deliberately imagine them), or involuntary (i.e. did they just come into your mind spontaneously), or could they be both? - The images were always voluntary. - The images were always involuntary. - The images could be either voluntary or involuntary. | These items were revised following reflection by the researcher, who determined that their original phrasing was too closed and potentially leading when presented separately. Combining them into a single multiple-choice item allowed respondents to indicate experiencing both voluntary and involuntary images and minimised bias in questioning. |
| Some text was added to the AAT flowchart to clarify to the interviewer that certain questions can be skipped if they are complete repeats of other questions, since the information has already been written down for the other question. | This aimed to improve the efficiency of AAT completion. |
| Interviewer instructions were added to the flowchart to instruct the interviewer to provide a couple of examples to illustrate what is meant by the ‘ability to access each goal’ items, when asking these questions. | These instructions were added to ensure that respondents fully understand the question before answering. |
| Some text was added to the first page of the AAT flowchart, providing further clarification to the interviewer instructions. | This aimed to provide further clarity to the interviewer on various aspects of AAT completion. |
| In both AAT sections, for the item asking respondents to list their most important goals, the interviewer text (which is read to the respondent before asking them to answer the question) was amended to state that these goals can include long or short-term goals. | This extra text was added because feedback from participants in the current study indicated the importance of short-term goals for some people during suicidal crises. Encouraging respondents to include short-term goals where applicable ensured that these potentially important goals would not be missed. |
| *Note.* The first set of AAT refinements followed feedback from the initial cognitive interviews in the current study; the second set of refinements followed feedback from the subsequent cognitive interviews. | |
